# METTL16-mediated m^6^A modification of MSMO1 modulates cholesterol metabolism and activates MAPK-p38/NF-κB signaling in colorectal cancer

**DOI:** 10.1186/s13046-026-03690-x

**Published:** 2026-03-17

**Authors:** Yongheng Zhao, Tingyue Gong, Hao Li, Haiping Lin, Minhao Yu, Yang Luo, Ming Zhong, Jun Qin

**Affiliations:** 1 Department of Gastrointestinal Surgery, Renji Hospital, Shanghai Jiao Tong University School of Medicine, Shanghai, China; 2 Department of General Surgery, Shanghai Pudong New Aera Gongli Hospital, Shanghai, China; 3 Department of General Surgery, Jinhua Central Hospital, Teaching Hospital of Mathematical Medicine College, Zhejiang Normal University, Zhejiang, China; 4 Ningbo Hangzhou Bay Hospital (Ningbo Branch of Renji Hospital, Shanghai Jiao Tong University School of Medicine, Shanghai), Zhejiang, China

**Keywords:** Colorectal Cancer (CRC), N^6^-methyladenosine(m^6^A), METTL16, MSMO1, cholesterol, MAPK

## Abstract

**Background:**

N^6^-methyladenosine (m^6^A) is the most abundant post-transcriptional modification, and METTL16 has recently emerged as a pivotal m^6^A methyltransferase in cancer. Cholesterol metabolic reprogramming and aberrant MAPK signaling sustain the malignant initiation and progression of colorectal cancer (CRC). However, whether - and how - m^6^A regulation, particularly by METTL16, interfaces with cholesterol metabolism and downstream oncogenic signaling in CRC remains unknown.

**Methods:**

Differential expression of METTL16 in CRC was identified through bioinformatic analyses and validated by qRT-PCR, immunoblotting, and immunohistochemistry (IHC). The functional role of METTL16 in CRC progression was examined using in *vitro* assays and xenograft models. To identify downstream targets, RNA-seq and MeRIP-seq were performed, revealing MSMO1 as a METTL16-regulated gene. The mechanistic basis of the METTL16–MSMO1 axis was investigated through immunoprecipitation-Mass Spectrometry (IP-MS), co-immunoprecipitation (Co-IP), RNA immunoprecipitation (RIP), MeRIP-qPCR, and RNA stability assays. Cholesterol metabolism analyses were conducted to further characterize the metabolic consequences of METTL16–MSMO1 regulation.

**Results:**

METTL16 was significantly upregulated in CRC and correlated with poor clinical outcomes. Mechanistically, METTL16 enhanced m^6^A modification of MSMO1, stabilizing its transcript via IGF2BP2 and disrupting intracellular cholesterol homeostasis, which triggered ER stress and activated MAPK-p38/ NF-κB signaling by promoting TAK1/TAB complex formation and TAK1 autophosphorylation, thereby driving CRC progression. Additionally, elevated cholesterol levels further reshaped global m^6^A methylation patterns and altered methyltransferase expression, suggesting a reciprocal feedback loop between cholesterol metabolism and epigenetic regulation.

**Conclusions:**

These findings underscore the critical role of the METTL16–MSMO1 axis in driving cholesterol metabolic reprogramming that fuels MAPK-p38/NF-κB oncogenic signaling in CRC, highlighting promising biomarkers and therapeutic targets for improved disease management.

**Supplementary Information:**

The online version contains supplementary material available at 10.1186/s13046-026-03690-x.

## Introduction

 Colorectal cancer (CRC) is the third most commonly diagnosed malignancy and the second leading cause of cancer-related mortality worldwide, with an estimated 1.9 million new cases and more than 930,000 deaths reported in 2022 [1]. Current standard treatments for CRC involve a multidisciplinary strategy that integrates curative surgery, radiotherapy, chemotherapy, targeted therapy, and immunotherapy [2, 3]. Despite advances in screening programs and therapeutic strategies, the 5-year survival rate for patients with advanced or metastatic CRC remains below 15%, largely due to tumor heterogeneity and therapeutic resistance [4]. Therefore, elucidating the molecular mechanisms that drive CRC initiation and progression is essential for the development of more effective treatment strategies.

RNA modifications have emerged as critical regulators of gene expression, among which N^6^-methyladenosine (m^6^A) represents the most prevalent internal post-transcriptional modification of RNA. m^6^A participates in nearly all aspects of RNA metabolism, including mRNA stability, translation, and splicing [5, 6]. As a dynamic and reversible epigenetic process, m^6^A is installed by methyltransferases—referred to as “writers,” such as METTL3, METTL14, and METTL16—and removed by the demethylases FTO and ALKBH5. m^6^A-binding proteins, or “readers,” including members of the IGF2BP family, recognize and bind m^6^A motifs to regulate the fate and function of target transcripts [7, 8]. Accumulating evidence indicates that dysregulated m^6^A modification contributes to tumor initiation and malignant progression [9]. METTL16, a relatively recently characterized m^6^A methyltransferase, has emerged as an important regulator of gene expression through RNA methylation–dependent mechanisms. Increasing evidence indicates that METTL16 contributes to tumorigenesis in multiple cancer types by modulating RNA stability, translation efficiency, and cellular metabolic programs [10–12]. In CRC, METTL16 has been reported to promote tumor progression by regulating glycolytic activity and lipid metabolic reprogramming [13, 14], highlighting its role in metabolic adaptation during malignant transformation. Nevertheless, while these studies established METTL16 as a metabolic regulator, the precise molecular pathways linking METTL16-mediated epi-transcriptomic regulation to specific lipid metabolic processes—particularly cholesterol homeostasis—remain insufficiently understood.

Deregulation of cellular metabolism, a prominent hallmark of cancer, profoundly facilitates tumor initiation and malignant progression [15]. Cholesterol, a major component of the cell membrane, is essential for maintaining cellular integrity and modulating the biophysical properties of lipid rafts [16]. In recent years, growing evidence has demonstrated that cholesterol metabolism plays a critical role in tumor development [17–19]. Notably, approximately 70–80% of solid tumors exhibit elevated endogenous cholesterol synthesis. Moreover, cholesterol has been shown to activate key oncogenic pathways, including MAPK and NF-κB signaling, and fluctuations in intracellular cholesterol levels can directly modulate MAPK activity, underscoring a close interplay between cholesterol metabolism and tumor-promoting signaling [20].

Methylsterol monooxygenase 1 (MSMO1), also known as sterol-C4-methyl oxidase-like (SC4MOL), catalyzes the demethylation of C4-methylated sterol intermediates and plays a pivotal role in maintaining intracellular cholesterol homeostasis, which is crucial for membrane integrity, lipid-raft formation, and diverse signaling processes in mammalian cells [21]. Dysregulated cholesterol metabolism has emerged as a hallmark of cancer, and accumulating evidence indicates that MSMO1 contributes to tumorigenesis in multiple malignancies. Elevated MSMO1 expression has been associated with the progression of esophageal, cervical, and breast cancers [22–24]. In addition, inhibition or genetic depletion of MSMO1 leads to the accumulation of sterol intermediates, activation of liver X receptor (LXR) signaling, and suppression of EGFR/KRAS-dependent tumor growth. Such inhibition also enhances EGFR internalization and degradation, thereby improving the therapeutic response to EGFR-targeted agents [25, 26]. However, despite these advances, the mechanistic role of MSMO1 in CRC remains largely undefined.

In this study, we identify a novel regulatory axis in CRC in which METTL16 promotes MSMO1 expression via IGF2BP2-dependent translation, linking m^6^A modification to cholesterol metabolism. We further demonstrate that MSMO1 directly interacts with TAK1, enhancing its autophosphorylation and contributing to the cascade activation of MAPK-p38 and NF-κB pathways, which drive CRC progression. Our findings unveil a previously unrecognized METTL16–MSMO1–MAPK/NF-κB signaling axis that couples metabolic remodeling with pro-inflammatory oncogenic signaling, offering potential therapeutic implications.

## Materials and methods

### Patient samples

Tissue samples were derived from surgically removed tissues of CRC inpatients at the Department of Gastrointestinal Surgery of Renji Hospital (Shanghai, China). Correlation between METTL16 expression and different clinical characteristics are shown in Supplementary Table 3. Written informed consent was provided by all patients. The acquisition and use of tumor specimens were approved by the Ethics Committee of Renji Hospital, School of Medicine, Shanghai Jiao Tong University (Approval number: LY2023-016-B).

### Nude mouse xenograft model

Male BALB/c nude mice (4 weeks old) were purchased from Gem Pharmatech (Nanjing, China) and housed with free access to food and water. DLD1 or RKO cells stably transfected with the indicated virus (3 × 10⁶ cells per mouse) were subcutaneously injected into the right flank of the mice (*n* = 5). Tumor volume was measured every five days and calculated using the formula 0.5 × a² × b, where a and b represent the short and long diameters, respectively. On day 28, the mice were euthanized, and the tumors were excised, weighed, fixed, and embedded for IHC analysis. All animal procedures were approved by the Experimental Animal Welfare and Ethics Committee of Renji Hospital, Shanghai Jiao Tong University School of Medicine, and conducted in accordance with relevant ethical guidelines (Approval number: m20241002).

### Immunohistochemistry (IHC) staining and analysis

Formalin-fixed, paraffin-embedded tissue Sect.  (3 μm) were subjected to IHC using a Leica BOND-MAX automated stainer (Leica Biosystems, Germany) according to the manufacturer’s instruction. Briefly, after deparaffinization, antigen retrieval, and blocking, sections were incubated with primary antibodies at 4 °C overnight, followed by secondary antibodies and DAB visualization. Staining intensity (negative: 0; weak: 1; moderate: 2; strong: 3) and percentage of positive stained (< 10%: 1; 10%-40%: 2; 40%-70%: 3; >70%: 4) were scored by two independent pathologists.

### Cells cultures and reagents

The normal human colonic epithelial cell line FHC and CRC cell lines HCT116, HCT15, HT29, DLD1, SW620, SW480, RKO were obtained from the Cell Repository of the Chinese Academy of Sciences (Shanghai, China). Cells were cultured in RPMI-1640, McCoy’s 5 A or DMEM medium supplemented with 10% fetal bovine serum (FBS) (#F0193, Sigma, USA). All cell lines were maintained in a humidified incubator at 37 °C in an atmosphere containing 5% CO_2_. Chemical compounds: SB203580 (10µM, p38 MAPK inhibitor, #HY-10256, MCE); Cholesterol (20µM, #HY-N0322, MCE); ER stress inhibitor: GSK2606414 (20µM, # HY-18072, MCE) and GSK2656157 (1µM, # HY-13820, MCE).

### Plasmid construction and transfection

All plasmids were purchased from Tsingke (Beijing, China), and their sequences are listed in Supplementary Table 2. Lentiviruses were generated by co-transfecting the knockdown or overexpression vectors with pMD2.G and psPAX2 into 293T cells using Lipofectamine 3000 (#L3000150, Invitrogen, USA). CRC cells were subsequently stably transduced with lentiviruses targeting METTL16 knockdown (shMETTL16), METTL16 overexpression (OE-METTL16/METTL16-WT), METTL16 mutant (METTL16-DM or METTL16-PP185/186AA) [27], MSMO1 knockdown (shMSMO1), MSMO1 overexpression (OE-MSMO1), IGF2BP2 knockdown (shIGF2BP2), or negative control (shNC, Ctrl) in the presence of polybrene (#40804ES76, YEASEN, China).

### Immunoblotting (IB)

Cells were lysed in RIPA buffer (#P0013K, Beyotime, China) supplemented with a protease inhibitor cocktail (#HY-K0011, MCE, USA) and a phosphatase inhibitor cocktail III (#HY-K0023, MCE, USA) to extract total cellular proteins. Protein concentration was measured using a BCA Protein Quantification Kit (#20201ES76, YEASEN, China). Equal amounts of protein were separated by SDS-PAGE (#PG222, EpiZyme, China) and transferred to PVDF membranes (#IPVH00010, Merck Millipore, Germany). The membranes were blocked with Protein Free Rapid Blocking Buffer (#PS108P, EpiZyme, China) at 25 °C for 1 h, followed by incubation with primary antibodies overnight at 4 °C. After washing, the membranes were incubated with appropriate secondary antibodies for 1 h at 25 °C. Protein bands were visualized using the Amersham Imager 600 (GE, USA). The primary antibodies used are listed in Supplementary Table 1.

### Real-time quantitative reverse transcription PCR (qRT‐PCR)

Total RNA was extracted from cells or tissues using TRIzol reagent (#12183555, Invitrogen, USA). cDNA was synthesized from RNA using the Hifair^®^ III 1st Strand cDNA Synthesis Kit (#11139ES60, YEASEN, China). Quantitative PCR was performed using the Hieff^®^ qPCR SYBR Green Master Mix (#11203ES03, YEASEN, China), with GAPDH or β-actin as the internal control. Primer sequences are provided in Supplementary Table 2.

### MeRIP-qPCR/Gene-specific m^6^A qPCR

m^6^A RNA immunoprecipitation (MeRIP) was performed using the Magna MeRIP m^6^A kit (#17-10499, Sigma, USA) according to the manufacturer’s instructions. Briefly, total RNA was extracted and then fragmented using RNA fragmentation reagents. One-tenth of the fragmented RNA was preserved as the input control. The remaining RNA was incubated with 5 µg of anti-m⁶A antibody or control IgG pre-conjugated to protein A/G magnetic beads in 500 µL IP buffer supplemented with 100 units of RNase inhibitor overnight at 4 °C. The immunoprecipitated complexes were treated with proteinase K at 52 °C for 1 h. Finally, purified RNA was then subjected to reverse transcription followed by qPCR. Primers sequences are listed in Supplementary Table 2. Relative m^6^A enrichment was normalized to the input using the following calculation: %Inupt = 2 ^(−Ct [RIP, normalized])^; Ct [RIP, normalized] = Ct [RIP] – (Ct [Input] – log_2_(Input Dilution Factor)), Input Dilution Factor = 10.

### Cell proliferation assays

For the cell proliferation assay, control and transfected cells were seeded into 96-well plates at a density of 1,000 cells per well, with five replicates per group. Cell viability was assessed at different time point (D1, D2, D3, D4, D5) using the Cell Counting Kit-8 (CCK-8) (#C0005, TargetMoI, USA) according to the manufacturer’s instructions. Briefly, 10 µL of CCK-8 reagent was added to each well and incubated at 37 °C for 2 h. The absorbance at 450 nm was then measured using a microplate reader.

### Colony formation assay

For colony formation assays, 800–1200 control or transfected cells in the logarithmic growth phase were seeded into six-well plates in triplicate and cultured for 10–14 days under standard conditions. The culture medium was replaced every 3–4 days. At the endpoint, colonies were gently washed twice with PBS, fixed with 4% paraformaldehyde (#G1101, Servicebio, China) for 20 min, and stained with Crystal Violet Staining Solution (#C0121, Beyotime, China) for an additional 20 min. Excess dye was removed by rinsing with tap water, and plates were air-dried. Colonies containing more than 50 cells were counted using ImageJ software.

### Transwell migration and invasion assay

Cell migration and invasion were evaluated using 24-well Transwell inserts (8-µm pore size; #CLS3422, Corning, USA). To eliminate potential interference from differential proliferation between groups, cells were pretreated with Mitomycin C (10 µg/mL) for 2 h to inhibit DNA replication and cell division prior to the assay. After thorough washing with PBS to remove residual drug, viable cells were counted using trypan blue exclusion, and equal numbers of cells (2.5 × 10⁴ per well) were resuspended in 200 µL of serum-free medium and seeded into the upper chambers.

For invasion assays, the upper chambers were precoated with Matrigel (#354234, BD Bioscience, USA) according to the manufacturer’s protocol. The lower chambers were filled with 500 µL of medium supplemented with 20% fetal bovine serum as a chemoattractant.

Cells were incubated for 48 h at 37 °C in a humidified atmosphere containing 5% CO₂. After incubation, non-migrated or non-invaded cells on the upper surface of the membrane were gently removed using cotton swabs. Cells that had migrated or invaded to the lower surface were fixed with 4% paraformaldehyde (#G1101, Servicebio, China) and stained with Crystal Violet Staining Solution (#C0121, Beyotime, China). Stained cells were visualized and imaged under an Olympus microscope, and representative fields were captured for quantitative analysis.

### RNA m^6^A dot blot assay

Total RNA extracted from cells was denatured at 95 °C for 3 min. Varying amounts of mRNA (100, 200, or 400 ng) were then spotted onto a nitrocellulose membrane (#41105339, Merck, Germany) and cross-linked under UV light for 2 h. The membranes were subsequently blocked with Protein Free Rapid Blocking Buffer (#PS108P, EpiZyme, China) for 1 h and incubated overnight at 4 °C with an anti-m⁶A antibody (#ab284130, Abcam, UK). After washing, the membranes were incubated with a secondary antibody for 1 h at room temperature. Signals were detected using the Amersham Imager 600 (GE, USA). Finally, membranes were stained with methylene blue (#A610622, Sangon, China) to verify equal RNA loading.

### m^6^A quantitation

The m^6^A RNA Methylation Assay Kit (#ab185912, Abcam, UK) was used to quantify the global m^6^A levels in total RNA. Total RNA was extracted from paired tumor and adjacent normal tissues of CRC patients. For each sample, 200 ng of RNA, along with an m6A standard, was added to the assay wells, followed by incubation with the Capture, Detection and Enhancer Solutions. After incubation with Developer Solution for 10 min at room temperature in the dark, the reaction was terminated by adding Stop Solution once the positive control wells turned medium blue. The absorbance at 450 nm was measured using a microplate reader, and m^6^A levels were calculated based on the standard curve.

### RIP assay

The RIP Assay Kit (#P1801, Beyotime, China) was used to assess the interaction between METTL16 and MSMO1 mRNA. Cells were washed twice with PBS and lysed in Lysis Buffer. The lysates were centrifuged at 14,000 × g for 10 min, and the supernatants were collected. In parallel, Protein A/G agarose beads were incubated with either anti-METTL16 antibody (#ab313743, Abcam, UK) or control IgG antibody (#a7016, Beyotime, China) for 4 h at 4 °C. The antibody–bead complexes were collected by centrifugation at 1,000 × g for 1 min, followed by incubation with the cell lysate supernatants overnight at 4 °C with gentle rotation. RNA was eluted using Elution Buffer and purified with the RNA Isolation Kit (#R2006, Beyotime, China). The purified RNA was subsequently analyzed by qRT-PCR, and fold enrichment of the target gene relative to the IgG negative control was calculated. Primer sequences used for this study are listed in Supplementary Table 2.

### mRNA stability assay

Cells were treated with 5 µg/mL actinomycin D (#HY-17559, MCE, USA) to inhibit transcription. Total RNA was extracted at 0, 2, 4, and 6 h using TRIzol reagent (#12183555, Invitrogen, USA) after treatment, and the expression levels of target genes at each time point were quantified by qRT-PCR.

### Total cholesterol quantification

Total cholesterol quantification was performed using the Amplex Red Cholesterol and Cholesteryl Ester Quantification Assay Kit (#S0211, Beyotime, China) in accordance with the manufacturer’s instructions. Briefly, 1 × 10^6^ cells were collected and lysed in 200 µL of BeyoLysis™ Buffer A for Metabolic Assay. Subsequently, 10 µL of the resulting lysate was combined with the prepared cholesterol detection solution and incubated at 37 °C for 30 min in the dark. The absorbance at 570 nm was measured with a spectrophotometer, and cholesterol levels were calculated based on the obtained values.

### RNA-seq and MeRIP-seq

MeRIP sequencing and data analysis were performed by LC-Bio Technology Co., Ltd. (Hangzhou, China). Total RNA was extracted from three pairs of DLD1 cells transfected with shMETTL16-1 or shNC using TRIzol reagent (#12183555, Invitrogen, USA). RNA concentration and purity were determined using NanoDrop ND-1000 (NanoDrop, USA), and RNA integrity was assessed using the Bioanalyzer 2100 system (Agilent, USA). Poly(A) RNA was isolated from 50 µg of total RNA using Dynabeads Oligo (dT)25 (#61005, Thermo Fisher, USA) through two rounds of purification. The purified poly(A) RNA was fragmented using the Magnesium RNA Fragmentation Module (#e6150, NEB, USA) at 86 °C for 7 min. For m6A immunoprecipitation, fragmented RNA was incubated with an m^6^A-specific antibody (#202003, Synaptic Systems, Germany) in IP buffer (50 mM Tris-HCl, 750 mM NaCl, and 0.5% Igepal CA-630) at 4 °C for 2 h. The immunoprecipitated RNA was reverse-transcribed into cDNA, followed by library construction and paired-end sequencing (PE150) using the Illumina NovaSeq™ 6000 platform (LC-Bio, Hangzhou, China), according to the manufacturer’s instructions. After quality filtering, raw sequencing reads were aligned to the human reference genome using HISAT2 (v2.2.1). m^6^A peak calling and differential methylation analysis were conducted using the exomePeak R package. Peak annotation was performed with ANNOVAR, while motif enrichment analysis was conducted using HOMER (v4.1). The mapped reads were assembled using StringTie (v2.1.2) with default settings. Transcript abundance was quantified using StringTie and ballgown by calculating FPKM. Differential gene expression analysis between groups was conducted using DESeq2. Differentially expressed genes were subjected to GO and KEGG pathway enrichment analyses. GSEA was performed using GSEA software (v4.1.0) together with gene sets from Midge to evaluate the enrichment of specific GO terms and KEGG pathways between the two groups.

### Coimmunoprecipitation (Co-IP) and mass spectrometry (MS)

For the immunoprecipitation (IP) assay, DLD1 cells transfected with the empty vector or Flag-MSMO1 expression plasmid were lysed in IP lysis buffer. Monoclonal ANTI-FLAG^®^ M2, Clone M2 (#F1804, Sigma, USA) was incubated with the lysates and beads overnight at 4 °C and then washed and collected according to the manufacturer’s protocol of Immunoprecipitation Kit with Protein G Magnetic Beads(#P2177, Beyotime, China). Mass spectrometry was performed by SpecAlly (Wuhan, China). Briefly, all samples were analyzed using an UltiMate 3000 RSLCnano system coupled online to a Q Exactive HF mass spectrometer via a Nanospray Flex ion source (Thermo, USA). MS raw data were processed in MaxQuant (v2.1.2.0) using the Andromeda database search algorithm. Spectra flies were searched against the Human protein database (2025-01-13, 20421 entries). Proteins that could not be distinguished based on unique peptides were merged by MaxQuant into one protein group. Label-free protein quantification was performed using the MaxLFQ algorithm and “match-between-runs” was enabled. Search results were filtered with 1% FDR at both peptide and protein levels. Proteins with a fold change > 4 between bait IP and control were screened out as interactors of the bait protein. Enrichment analysis of interactors was performed using a two-sided hypergeometric test, and GO terms and KEGG pathways with *p* < 0.05 were considered significantly enriched.

For the Co-IP assay, western blotting was performed to determine protein levels. Cells were rinsed in PBS and then lysed in IP lysis buffer. The protein extracts were subsequently incubated with the Monoclonal ANTI-FLAG^®^ M2, Clone M2 (#F1804, Sigma, China), Myc-Tag mAb (#M20002, Abmart, China) and beads. The precipitated proteins were separated and detected by western blotting using the indicated antibodies. Finally, the blots were visualized using a chemiluminescence system.

### Bioinformatics analysis

The colorectal cancer datasets were downloaded from the Gene Expression Omnibus (GEO; accession number GSE110224) and The Cancer Genome Atlas (TCGA) databases. Bioinformatics analyses and data visualization were performed using R (v4.5.1) and GraphPad Prism (v10.5.0).

### Statistical analysis

For continuous variables, comparisons between two groups were performed using *Student’s t-*test or the *Wilcoxon rank-sum* test according to data distribution. For comparisons among three or more groups, ordinary *one-way or two-way ANOVA* or the *Kruskal–Wallis* test was applied as appropriate. Categorical variables were analyzed using the *Chi-square* test or *Fisher’s exact* test. Data are presented as mean ± SEM. Overall survival was estimated with the *Kaplan–Meier* method, and survival differences were assessed using the *log-rank* test. Statistical analyses were carried out using SPSS (v27.0.1.0), GraphPad Prism (v10.5.0) and R (v4.5.1). A two-sided *P* < 0.05 was considered statistically significant (*P < 0.05, **P < 0.01, ***P < 0.001, ****P < 0.0001).

## Results

### METTL16 overexpression and its prognostic value in CRC

**Fig. 1:**
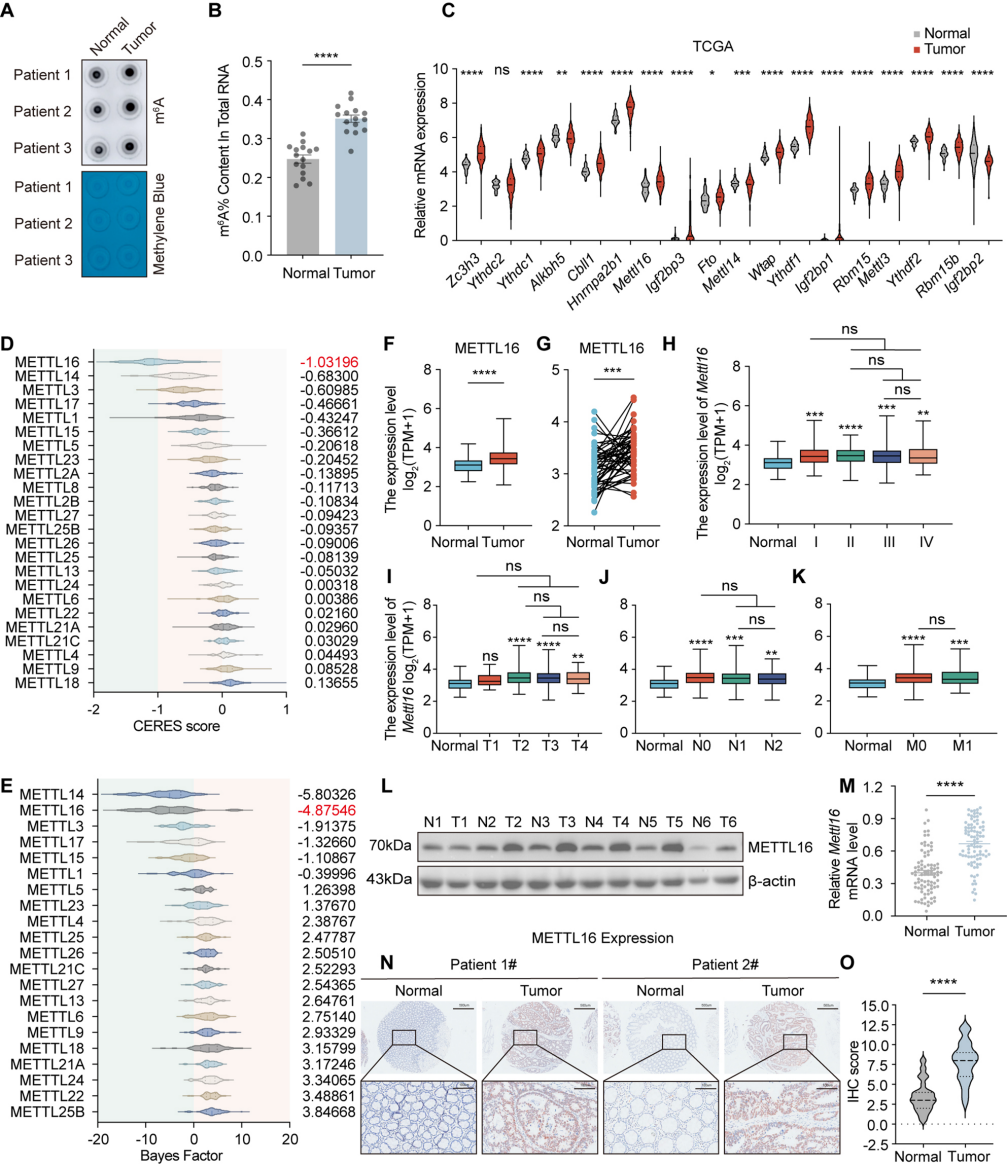
METTL16 overexpression and its prognostic value in CRC. **A** Dot blot analysis of mRNAs isolated from CRC tissues and paired normal intestinal mucosa using an anti-m6A antibody. Methylene blue staining was used as a loading control. Representative images are shown in the panel above. **B** The m6A RNA levels in 15 paired CRC tissues and normal intestinal mucosa were quantified using a colorimetric ELISA-like assay with the m6A RNA methylation quantification kit. **C** Relative mRNA expression levels of m6A regulatory factors in tumor and normal tissues from the TCGA-COAD/READ database. **D** CERES scores of METTL family members obtained from CRISPR-Cas9 knockout screening datasets across 63 human CRC cell lines. The raw data were retrieved from DepMap (https://depmap.org/portal/). CERES scores of 0 and -1 correspond to the median effects of non-essential genes and core essential genes, respectively. Lower CERES scores indicate greater dependency of the specific gene on cancer progression. The average CERES score for each METTL family member is presented on the right. **E** Violin plots depicting the Bayes Factor of a set of METTL family members from genome-scale CRISPR-Cas9 essentiality screens across 46 human CRC cell lines. The raw data were retrieved from (https://score.depmap.sanger.ac.uk/). The Creative Commons Public Domain Dedication waiver. The average Bayes Factor for each METTL family member is shown. **F** and **G** METTL16 expression in CRC tissues versus nonmatched (**F**) or matched (**G**) adjacent normal tissues from TCGA-COAD/READ datasets. **H**-**K** Association of Mettl16 mRNA expression with pathological stage (**H**), tumor size (**I**), lymph node metastasis (**J**), and distant metastasis (**K**) in CRC patients from the TCGA-COAD/READ database. Lymph node metastasis categories: N0 (no lymph node metastasis), N1 (nearby lymph node metastasis), N2 (distant lymph node metastasis). Metastasis categories: M0 (no distant metastasis), M1 (distant metastasis). **L** METTL16 protein levels were measured in CRC tissues and paired normal intestinal mucosal tissues by western blotting (*n*=6). **M** Mettl16 expression levels in 86 pairs of CRC tissues and matched adjacent non-cancerous intestinal tissues were measured by RT-qPCR. **N** Representative images of METTL16 IHC staining in CRC tissues and adjacent normal tissues. scale bars=500 μm, 100 μm. **O** IHC staining scores of METTL16 in CRC tissues and adjacent normal tissues. Results shown are the mean ± SEM (ns, non-significant; **p < 0.01, ***p < 0.001, ****p < 0.0001) of triplicate determination from three independent experiments

N^6^-methyladenosine (m^6^A) represents the most abundant internal modification within eukaryotic messenger RNA. Dysregulated m^6^A modification patterns have been identified in various malignancies and are strongly associated with oncogenic initiation and progression [9]. To elucidate the potential involvement of m^6^A modification in CRC, we first quantified global m^6^A methylation levels in CRC tissues and matched adjacent normal intestinal mucosa. A significant increase in total m^6^A modification was observed in tumor specimens compared with adjacent non-tumorous tissues (Fig. 1A and B), indicating that global m^6^A methylation is substantially elevated in clinical CRC samples relative to normal mucosa. Subsequently, transcriptomic profiling of key m^6^A regulators and METTL family members was performed using the TCGA-COAD/READ dataset (Fig. 1C; Supplementary Fig. 1A). Most m^6^A regulatory components exhibited distinct expression patterns between CRC and normal tissues, suggesting widespread dysregulation of the m^6^A machinery during colorectal tumorigenesis. By analyzing two independent large-scale genome-wide CRISPR–Cas9 knockout screening datasets, we identified METTL16 as the most critical METTL family gene required for the survival of CRC cells (Fig. 1D and E). Consistently, METTL16 mRNA expression was markedly elevated in CRC tissues compared with adjacent normal counterparts, as evidenced by analyses of both TCGA and GEO (GSE110224) datasets (Fig. 1F and G; Supplementary Fig. 1B).

Moreover, analysis of TCGA data revealed a strong positive association between METTL16 expression and unfavorable clinicopathologic parameters, including larger tumor size, lymph node metastasis, distant dissemination, and advanced clinical stage (Fig. 1H–K). In freshly obtained CRC specimens, METTL16 expression was significantly increased at both the mRNA and protein levels in tumor tissues compared with paired adjacent normal mucosa (Fig. 1L and M). Immunohistochemical staining further verified the elevated expression of METTL16 in CRC tissues (Fig. 1N and O). Furthermore, Kaplan–Meier survival analysis indicated that higher METTL16 expression was significantly correlated with poorer overall survival (OS) in CRC patients (Supplementary Fig. 1C). Collectively, these results demonstrate that METTL16 is markedly upregulated in CRC and closely associated with aggressive clinicopathologic features and poor patient prognosis, suggesting that METTL16 may serve as a potential oncogenic driver and prognostic biomarker in CRC.

### METTL16 enhances the malignant behavior of CRC cells *in vitro* and *in vivo*

**Fig. 2:**
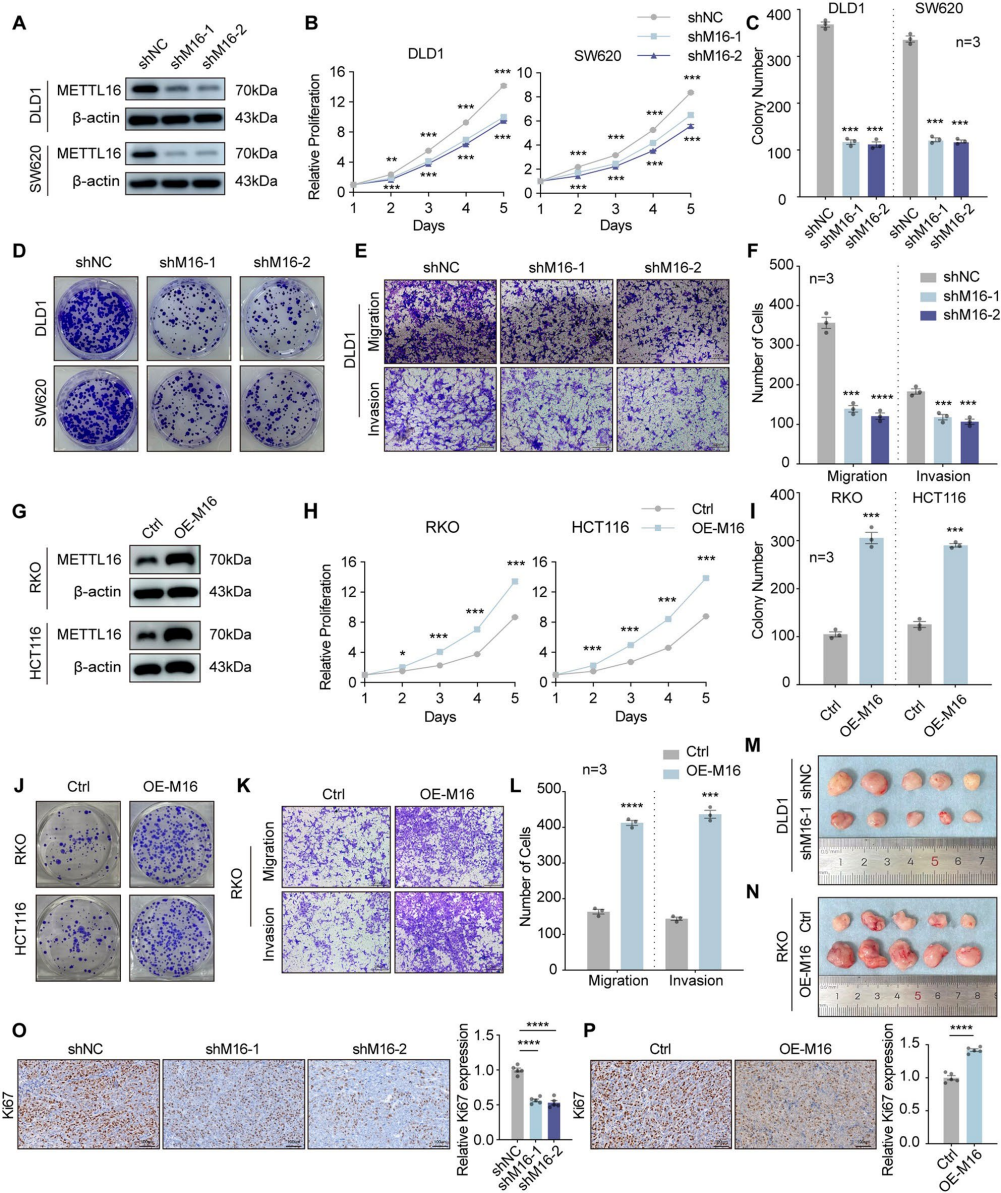
METTL16 enhances the malignant behavior of CRC cells in vitro and in vivo. **A** The protein levels of METTL16 in DLD1 and SW620 cells with METTL16 knockdown (shNC, shM16-1 and shM16-2) were measured by western blotting. **B** Cellular proliferation of DLD1 and SW620 cells with METTL16 knockdown or control was detected by CCK‐8 assay. **C** and **D** Colony formation assays were conducted to assess the proliferative capacity of DLD1 and SW620 cells following METTL16 knockdown. Representative images (**D**) and quantitative analyses (**C**) are shown. **E** and **F** Transwell assays were performed to detect the migrative and invasive capacity of DLD1 with METTL16 knockdown. Representative images (scale bars=200 μm, **E**) and quantification (**F**) of the cell migration and invasion assay results were shown. **G** The protein levels of METTL16 in RKO and HCT116 cells with METTL16 overexpression were measured by western blotting. **H** Cellular proliferation of RKO and HCT116 cells with METTL16 overexpression or control was detected by CCK‐8 assay. **I** and **J** Colony formation assays were performed to detect the proliferation of RKO and HCT116 cells with METTL16 overexpression. Representative images **J** and quantification **I** of results were shown. **K** and **L** Transwell assays were performed to detect the migrative and invasive capacity of RKO with METTL16 knockdown. Representative images (scale bars=200 μm, **K**) and quantification (**L**) of the cell migration and invasion assay results were shown. **M** and **N** Xenografts derived from DLD1‐shM16 cells **M** or RKO‐OEM16 cells (**N**) and relative controls (*n* = 10). **O** and **P** Ki67 IHC staining of paraffin‐embedded sections obtained from xenografts based on DLD1 cells with METTL16 knockdown (**O**) or RKO cells with METTL16 overexpression (**P**). Scale bars=100 μm. Results shown are the mean ± SEM (ns, non-significant; *p < 0.05, **p < 0.01, ***p < 0.001, ****p < 0.0001) of triplicate determination from three independent experiments

To elucidate the functional significance of METTL16 in CRC progression, we initially assessed its oncogenic potential. Both mRNA and protein levels of METTL16 were substantially higher in CRC cell lines than in normal colonic epithelial cells (Supplementary Fig. 2A and B). Subsequently, METTL16 expression was silenced in CRC cell lines (DLD1 and SW620) using two independent short hairpin RNAs (shMETTL16-1 and shMETTL16-2), as confirmed by both mRNA and protein analyses (Fig. 2A; Supplementary Fig. 2C and D). Functionally, silencing METTL16 markedly inhibited CRC cell proliferation, as evidenced by both CCK-8 and colony formation assays (Fig. 2B–D). In addition, METTL16 depletion significantly reduced the migratory and invasive capabilities of CRC cells (Fig. 2E and F; Supplementary Fig. 2G and H). Conversely, stable METTL16-overexpressing CRC cell lines (RKO and HCT116) were established using a pHBLV-METTL16 overexpression plasmid (OE-METTL16) (Fig. 2G; Supplementary Fig. 2E and F). As expected, enforced METTL16 expression significantly enhanced the clonogenic potential of CRC cells (Fig. 2H–J). Moreover, ectopic overexpression of METTL16 markedly promoted the migratory and invasive behavior of RKO and HCT116 cells (Fig. 2K and L; Supplementary Fig. 2I and J).

To further substantiate the role of METTL16 in CRC tumorigenesis in vivo, a subcutaneous xenograft mouse model was established. Tumors derived from METTL16-silenced CRC cells exhibited markedly reduced growth rates, as evidenced by decreased tumor volume and weight compared with control-derived xenografts (Fig. 2M; Supplementary Fig. 2K–M). Conversely, enforced METTL16 expression produced the opposite effect, leading to significantly enhanced tumor growth (Fig. 2N; Supplementary Fig. 2N and O). In addition, immunohistochemical staining revealed decreased expression of Ki-67, a proliferation marker, in tumor tissues from the METTL16-knockdown group compared with control tumors (Fig. 2O), whereas tumors derived from METTL16-overexpressing cells displayed a pronounced increase in Ki-67 staining intensity (Fig. 2P). Collectively, these results demonstrate that METTL16 facilitates CRC progression both in vitro and in vivo.

### METTL16 deficiency dampens the MAPK-p38 signaling pathway

**Fig. 3:**
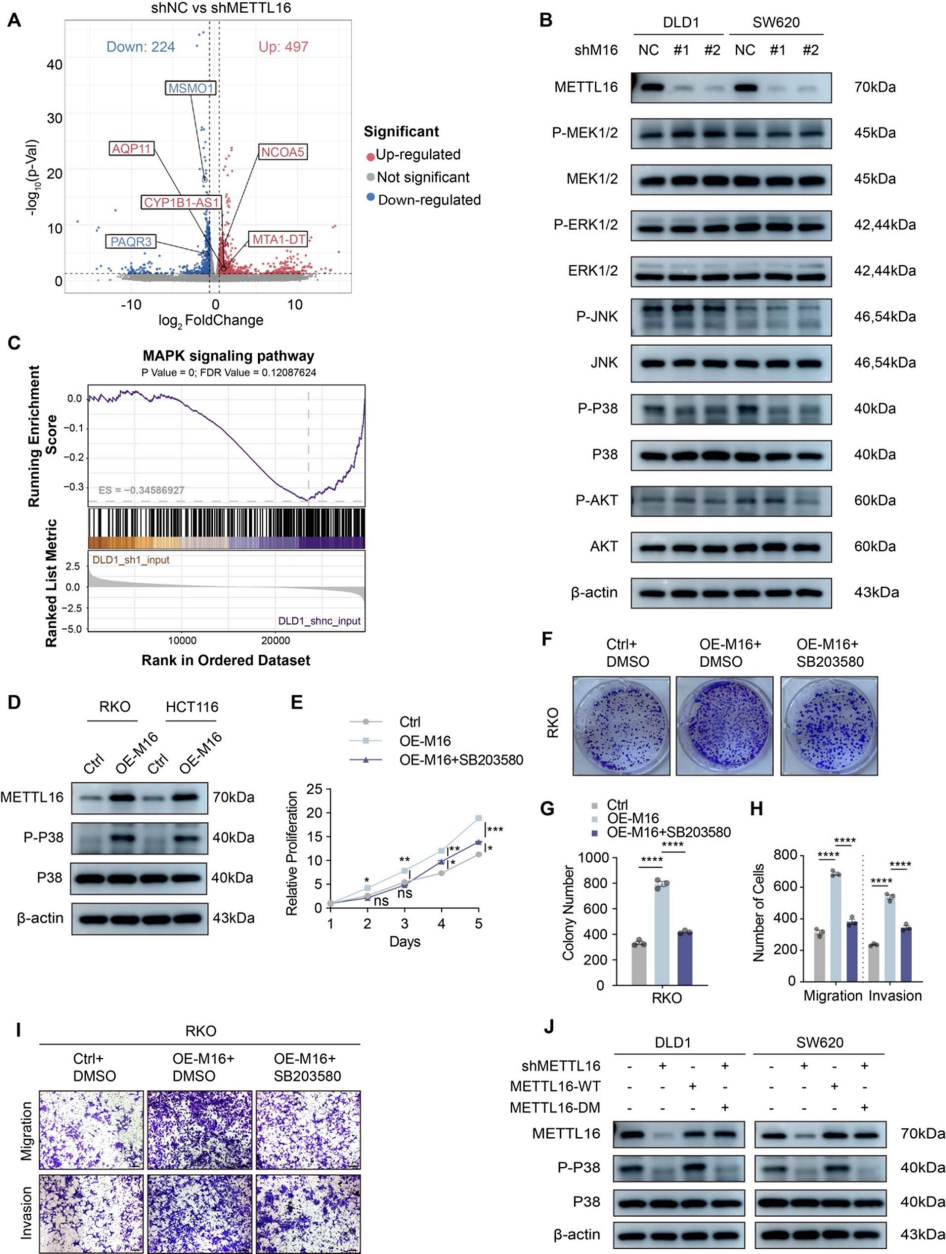
METTL16 deficiency dampens the MAPK-p38 signaling pathway. **A** RNA-seq volcano plot of differential gene expression after METTL16 knockdown in DLD1 cells |log2(fc)|>=1, *p *< 0.05. **B** Western blotting analysis was performed to determine the regulatory effects on MAPK/PI3K-AKT signaling pathways in control (shNC) and METTL16-silenced (shM16-1, shM16-2) CRC cells. **C** GSEA enrichment curve of the MAPK signaling pathway illustrating its correlation with METTL16 expression (NES = –1.699813, FDR = 0.12087624). **D** Western blotting showing the activation of MAPK-p38 in METTL16-overexpressing CRC cells. **E** CCK8 assay evaluating the proliferation of RKO cells following inhibition of the MAPK-p38 pathway using the p38 inhibitor SB203580. **F** and **G** Colony formation assay showing that inhibition of the MAPK-p38 pathway reduces the proliferative effect of METTL16 overexpression on RKO cells. Representative images (**F**) and quantification (**G**) of results were shown. **H** and **I** Transwell assay examining the effect of METTL16 overexpression on RKO migration and invasion following MAPK-p38 pathway inhibition. Representative images (scale bars=100 μm, **I**) and quantification (**H**) of results were shown. **J** Western blotting analysis showing the effect of METTL16 methyltransferase activity on the activation of the MAPK-p38 signaling pathway. Results shown are the mean ± SEM (ns, non-significant; *p < 0.05, **p < 0.01, ***p < 0.001, ****p < 0.0001) of triplicate determination from three independent experiments

To elucidate the molecular mechanisms underlying METTL16-driven CRC proliferation, RNA sequencing was performed on CRC cells with stable METTL16 knockdown and matched control cells. Transcriptome profiling revealed substantial alterations in gene expression, with 497 genes upregulated and 224 genes downregulated (Fig. 3A; Supplementary Fig. 3A and B). Kyoto Encyclopedia of Genes and Genomes (KEGG) enrichment analysis initially identified the PI3K/AKT signaling pathway among the top enriched pathways based on differentially expressed genes following METTL16 depletion (Supplementary Fig. 3C). Consistently, Gene Set Enrichment Analysis (GSEA) also demonstrated significant enrichment of PI3K/AKT-related gene sets (Supplementary Fig. 3D). Based on these bioinformatic findings, the functional status of the PI3K/AKT pathway was subsequently examined. However, in-vitro validation showed that neither total AKT protein levels nor its phosphorylation status was significantly altered upon METTL16 knockdown, indicating that transcriptomic enrichment of PI3K/AKT components did not translate into measurable changes in pathway activation at the protein level **(**Fig. 3B**)**. Extensive evidence has established the pivotal role of the p38/MAPK branch in mediating inflammatory responses and cellular stress signaling [28]. Upon activation, p38/MAPK regulates downstream transcriptional programs by modulating key inflammatory transcription factors such as NF-κB and ATF family members, thereby amplifying inflammatory gene expression and influencing tumor-associated inflammatory processes [20, 29, 30]. Although MAPK signaling was not the highest-ranked pathway in the KEGG analysis, it exhibited consistent and statistically significant enrichment in the GSEA, suggesting coordinated transcriptional regulation at the pathway level **(**Fig. 3C**)**.

Notably, silencing METTL16 in DLD1 and SW620 cells led to a significant reduction in p38 phosphorylation, with no changes observed in total p38 expression levels **(**Fig. 3B**)**. Conversely, ectopic overexpression of full-length METTL16 in RKO and HCT116 cells significantly enhanced p38 phosphorylation **(**Fig. 3D**)**. Moreover, treatment with the p38 inhibitor SB203580 effectively suppressed METTL16-induced CRC cell proliferation. Pharmacological inhibition of p38 significantly inhibited CRC cell proliferation, migration, and invasion, as assessed by CCK-8, colony formation, and transwell assays **(**Fig. 3E–I**)**. To investigate whether the methyltransferase activity of METTL16 is critical for activating the signaling pathway, we reintroduced either wild-type METTL16 (METTL16-WT) or a catalytically inactive mutant METTL16 (METTL16-DM) into METTL16 knockdown CRC cells. The restoration of wild-type METTL16 led to the recovery of p38 phosphorylation, a key event in the MAPK signaling cascade. In contrast, the catalytically inactive mutant METTL16 failed to restore p38 phosphorylation, suggesting that the methyltransferase activity of METTL16 is indispensable for activating this pathway **(**Fig. 3J**)**. Taken together, these findings demonstrate that METTL16 promotes CRC progression via activation of the MAPK-p38 signaling pathway in a methyltransferase activity-dependent manner.

### METTL16 facilitates *Msmo1* transcript stability in an m^**6**^A-IGF2BP2-dependent manner

**Fig. 4:**
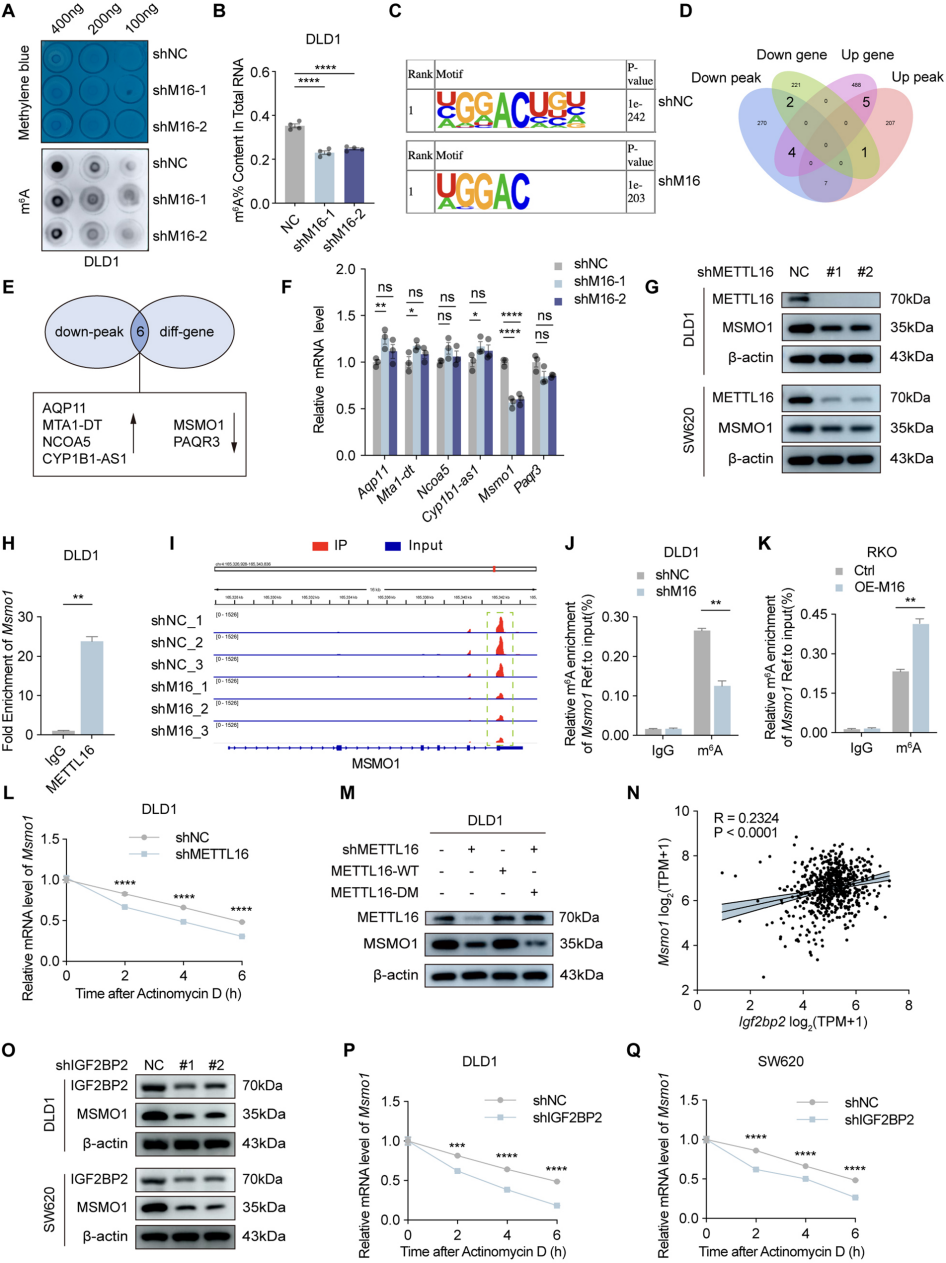
METTL16 facilitates *Msmo1* transcript stability in an m^6^A-IGF2BP2-dependent manner. **A** Dot blot analysis of mRNAs isolated from METTL16-knockdown (shM16-1, shM16-2) and control (shNC) DLD1 cells, with methylene blue staining as loading control. Representative images shown above. **B** The m6A RNA levels of METTL16 knockdown (shM16-1, or shM16-2) and control (shNC) DLD1 cells were detected by colorimetric ELISA-like assay via the m6A RNA methylation quantification kit. **C** Specific m6A motif analysis in stable shNC and shMETTL16 cell lines. **D** Venn diagram displays 12 differential genes were classified according to the level of mRNA and m6A peak in DLD1 cells with METTL16 knockdown. **E** 6 candidate target genes of METTL16 came from the intersection of RNA‐sequencing and MeRIP‐sequencing. **F** The 6 candidate target genes mRNA expression in DLD1 cells with METTL16 knockdown were detected by qRT‐PCR. **G** The MSMO1 protein expression in DLD1 and SW620 cells with METTL16 knockdown were detected by western blotting. **H** RIP-qPCR experiments identified whether Msmo1 mRNA binds with METTL16 in DLD1 cells. **I** The m6A peak visualization of m6A‐seq in Msmo1 transcripts in DLD1 cells with or without METTL16 knockdown was shown. **J** Relative m6A enrichment of Msmo1 mRNA in DLD1 cells with or without METTL16 knockdown was analyzed and normalized to input by using MeRIP‐qPCR. **K** Relative m6A enrichment of Msmo1 mRNA in RKO cells with or without METTL16 overexpression was analyzed and normalized to input by using MeRIP‐qPCR. **L** Treatment of DLD1 cells with actinomycin D (10 μg/ml) post METTL16 knockdown, and assessment of Msmo1 mRNA stability at specific times (0h, 2h, 4h, 6h) via qRT-PCR. **M** Western blot analysis showing the impact of METTL16 methyltranrase activity on MSMO1 protein levels in DLD1 cells. **N** Correlation between Igf2bp2 mRNA and Msmo1 mRNA. **O** Western blotting confirmed the expression of IGF2BP2 and MSMO1 protein levels following IGF2BP2 knockdown in DLD1 and SW620 cells. **P** and **Q** Treatment of DLD1 (**P**) and SW620 cells (**Q**) with actinomycin D (10 μg/ml) post IGF2BP2 knockdown, and assessment of Msmo1 mRNA stability at specific times (0h, 2h, 4h, 6h) via qRT-PCR. Results shown are the mean ± SEM (ns, non-significant; *p < 0.05, **p < 0.01, ***p < 0.001, ****p < 0.0001) of triplicate determination from three independent experiments

METTL16, functioning as an RNA methyltransferase, plays a pivotal role in regulating RNA splicing, nuclear export, stability, and translation, thereby influencing diverse aspects of cancer progression [12, 31]. To explore its functional relevance in CRC, we examined the impact of METTL16 depletion on global m^6^A methylation levels. Both dot blot analysis and quantitative m^6^A RNA methylation assays revealed a pronounced reduction in total m^6^A levels in CRC cells transduced with shMETTL16 compared with the shNC control group, underscoring the essential role of METTL16 in maintaining m^6^A homeostasis in CRC (Fig. 4A and B). To identify the downstream effectors regulated by METTL16-mediated m^6^A modification, we performed methylated RNA immunoprecipitation sequencing (MeRIP-seq) to profile the global methylome in DLD1 cells with stable METTL16 knockdown and in matched control cells. Comparative analysis revealed widespread alterations in m^6^A methylation patterns upon METTL16 depletion, with 236 peaks upregulated and 294 peaks downregulated relative to the control group (Supplementary Fig. 4A). As shown in Fig. 4C, canonical m^6^A consensus motifs (RRACH) were identified in both groups. The m^6^A peaks were mainly enriched in the 3′-UTR and around the stop codon regions in both shNC and shMETTL16 cells (Supplementary Fig. 4B-D). Similarly, differentially modified peaks were predominantly localized within the 3′-UTR and stop codon regions (Supplementary Fig. 4E). By integrating the results from both sequencing analyses, we identified 12 overlapping genes between the differentially expressed genes and differential m^6^A peaks (Fig. 4D). Among the downregulated m^6^A peaks, the expression of six genes —CYP1B1-AS1, AQP11, MTA1-DT, NCOA5, MSMO1 and PAQR3 —was significantly altered (Fig. 4E; Supplementary Fig. 4F).

To validate the downstream gene candidates, qRT-PCR was performed to assess the mRNA expression levels of the six genes identified above. Among these candidates, only MSMO1 exhibited a consistent expression pattern, being significantly downregulated upon METTL16 depletion and upregulated upon METTL16 overexpression (Fig. 4F; Supplementary Fig. 5A). Consistently, METTL16 positively regulated MSMO1 protein abundance (Fig. 4G; Supplementary Fig. 5B). To confirm a direct interaction between METTL16 and MSMO1 transcripts, analysis of a previously published RNA immunoprecipitation sequencing (RIP-seq) dataset (GEO: GSE156797) [27] revealed a pronounced enrichment of *Msmo1* mRNA in the METTL16 immunoprecipitates, with an IP/Input ratio > 9. Notably, the enrichment level of MSMO1 exceeded that observed for several previously reported METTL16 targets, including BCAT1, BCAT2, SOGA1 and TM7SF2, suggesting a strong and specific binding affinity of METTL16 toward the *Msmo1* transcript (Supplementary Fig. 5C). This finding was further validated by RIP-qPCR, which confirmed the specific binding of METTL16 to *Msmo1* mRNA (Fig. 4H). To identify potential m^6^A modification sites on *Msmo1* mRNA, we utilized the SRAMP prediction platform [32], which predicted several potential m^6^A sites located near the 3′-UTR and stop codon regions (Supplementary Fig. 5D and E). Consistently, inspection of our MeRIP-seq dataset using Integrative Genomics Viewer (IGV) visualization revealed a decreased m^6^A peak in the MSMO1 transcript following METTL16 knockdown (Fig. 4I), further supporting that MSMO1 is a direct downstream target of METTL16-dependent m^6^A modification. MeRIP-qPCR analysis revealed that the m^6^A-modified *Msmo1* mRNA levels were significantly reduced upon METTL16 knockdown, whereas METTL16 overexpression led to a marked increase in m^6^A-modified *Msmo1* mRNA (Fig. 4J and K). Given that m^6^A modification influences RNA stability and consequently affects target RNA expression [33], RNA stability assays confirmed that silencing METTL16 decreased the stability of MSMO1 mRNA (Fig. 4L). Furthermore, Western blot analysis demonstrated that METTL16 regulates MSMO1 protein expression in a methyltransferase activity-dependent manner (Fig. 4M; Supplementary Fig. 5F).

In m^6^A-mediated post-transcriptional regulation, reader proteins interpret methylation marks to dictate transcript fate, thereby modulating RNA stability, translation, or decay. Among established m^6^A readers, the IGF2BP family has been defined as a class of stabilizing factors that preferentially bind methylated transcripts and protect them from degradation in cancer-associated contexts [7]. Given that METTL16 depletion reduced both m^6^A enrichment and the stability of *Msmo1* mRNA, we hypothesized that a stabilization-type reader is likely responsible for mediating this regulatory effect. To identify the most relevant candidate in CRC, we performed correlation analyses using TCGA-COAD/READ datasets. Notably, IGF2BP2 exhibited the strongest positive association with MSMO1 expression among major m^6^A readers (Fig. 4N; Supplementary Fig. 5G and H), providing a clinically supported basis for prioritization.

To determine whether IGF2BP2 functions as the m^6^A reader of *Msmo1* mRNA, we performed IGF2BP2 knockdown experiments. Silencing IGF2BP2 led to a pronounced decrease in both *Msmo1* mRNA and MSMO1 protein levels (Fig. 4O; Supplementary Fig. 5I). Consistently, RNA stability assays confirmed that IGF2BP2 knockdown reduced *Msmo1* mRNA stability (Fig. 4P and Q). Collectively, these findings indicate that METTL16-mediated m^6^A modification sustains MSMO1 expression by promoting IGF2BP2-dependent stabilization of *Msmo1* mRNA.

### METTL16 accelerates the CRC malignant process by elevating MSMO1 expression

To determine whether MSMO1 is consistently dysregulated across CRC cell models and to evaluate its potential dependency pattern, we first examined MSMO1 expression across a panel of CRC cell lines. As shown in Supplementary Fig. 6A, MSMO1 expression levels varied substantially among different CRC cell lines. Notably, although several CRC models exhibited increased MSMO1 expression compared with the normal colonic epithelial cell line FHC, others displayed comparable or even lower expression levels relative to FHC. These findings indicate that MSMO1 upregulation is not uniformly observed across all CRC cell lines. Consistent with this observation, analysis of the DepMap CRISPR-Cas9 screening dataset demonstrated that MSMO1 generally exhibited CERES scores (or Bayes factors) close to zero in most models (Supplementary Fig. 6B). This result suggests that MSMO1 is not classified as a broadly essential gene across all CRC cell lines within this large-scale screening dataset.

**Fig. 5:**
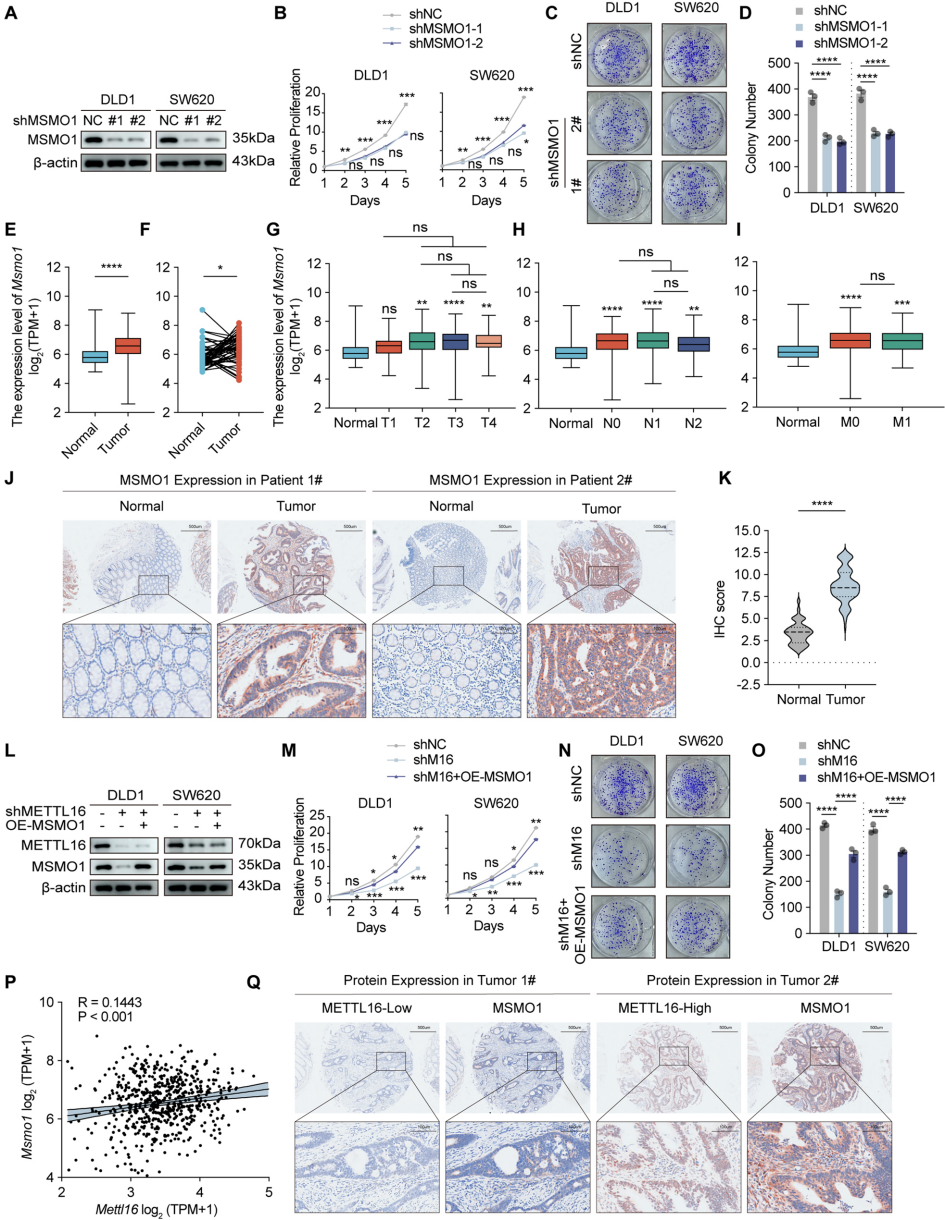
METTL16 accelerates the CRC malignant process by elevating MSMO1 expression. **A** The protein levels of MSMO1 in DLD1 and SW620 cells with MSMO1 knockdown (shNC, sh-1 and sh-2) were measured by western blotting. **B** Cellular proliferation of DLD1 and SW620 cells with MSMO1 knockdown or control was detected by CCK‐8 assay. **C** and **D** Colony formation assays were performed to detect the proliferation of DLD1 and SW620 cells with MSMO1 knockdown. Representative images (**C**) and quantification (**D**) of results were shown. **E** and **F** MSMO1 expression in CRC tissues versus nonmatched (**E**) or matched (**F**) adjacent normal tissues from TCGA-COAD/READ datasets. **G**-**I** Association of Msmo1 mRNA expression with tumor size (**G**), lymph node metastasis (**H**), and distant metastasis (**I**) in CRC patients in TCGA-COAD/READ database. **J** and **K** Representative images of MSMO1 IHC staining in CRC tissues and adjacent normal tissues (**J**). Quantification of IHC staining scores of MSMO1 in CRC tissues and adjacent normal tissues (**K**). Scale bars=100 μm, 500μm. **L**-**O** Forced overexpression of MSMO1 rescues the deficiency of CRC cell proliferation and colony formation induced by METTL16 depletion. MSMO1 expression construct was transfected in DLD1 and SW620 cells for 48 h and MSMO1 protein expression was determined by immunoblotting (**L**). CCK8 cell proliferation (**M**) and colony formation assay was performed in DLD1 and SW620 cells transfected with MSMO1 overexpression or control construct. Representative images (**N**) and quantification (**O**) of colony formation assay were shown. **P** Correlation analysis between Mettl16 and Msmo1 mRNA expression. **Q** Representative images of METTL16 and MSMO1 IHC staining from CRC tissues. Tumor 1# is representative of a patient with METTL16-low CRC. Tumor 2# is representative of a patient with METTL16-high CRC. Scale bars=100 μm, 500μm. Results shown are the mean ± SEM (ns, non-significant; *p < 0.05, **p < 0.01, ***p < 0.001, ****p < 0.0001) of triplicate determination from three independent experiments

To further evaluate the functional relevance of MSMO1 in CRC progression, we performed loss-of-function assays in DLD1 and SW620 cells, which exhibited relatively high endogenous MSMO1 expression (Supplementary Fig. 6A). Notably, these two cell lines also showed elevated METTL16 expression in our previous analyses, ensuring consistency within our experimental framework. Silencing MSMO1 significantly suppressed cell proliferation and colony formation in both DLD1 and SW620 cells (Fig. 5A–D). Despite the variability observed at the cell-line level, transcriptomic analysis of the TCGA-COAD/READ cohorts revealed that MSMO1 mRNA expression was significantly upregulated in colorectal tumor tissues compared with adjacent normal tissues (Fig. 5E and F). Furthermore, elevated MSMO1 expression was positively associated with adverse clinicopathological features, including increased tumor size, lymphatic metastasis, distant dissemination, and advanced clinical stage (Fig. 5G–I; Supplementary Fig. 6C). Consistently, immunohistochemical staining confirmed enhanced MSMO1 protein expression in CRC specimens (Fig. 5J and K). Collectively, although MSMO1 does not exhibit uniform dependency across CRC cell lines in large-scale CRISPR screening datasets, our clinical observations together with functional validation experiments support a potential role for MSMO1 in promoting CRC progression under specific experimental conditions.

To further substantiate the potential functional relevance of the METTL16–m^6^A–MSMO1 regulatory axis in CRC progression, both in vitro cellular assays and in vivo xenograft models were performed. In vitro experiments demonstrated that METTL16 silencing markedly suppressed CRC cell proliferation, colony formation, migration, and invasion, whereas enforced expression of MSMO1 effectively rescued these impaired oncogenic phenotypes (Fig. 5L–O). Consistently, in vivo xenograft analyses revealed that METTL16 knockdown significantly inhibited tumor growth, while MSMO1 overexpression partially reversed this inhibitory effect (Supplementary Fig. 6E–H). Furthermore, transcriptomic profiling of TCGA-COAD/READ datasets demonstrated a significant positive correlation between METTL16 and MSMO1 expression levels in CRC (Fig. 5P), which was further corroborated by immunohistochemical analysis of clinical colorectal tumor specimens (Fig. 5Q; Supplementary Fig. 6D). Collectively, these results identify MSMO1 as a functionally critical downstream target of METTL16 in CRC and reveal that the METTL16/MSMO1 axis is clinically associated with poor prognosis in CRC patients.

### MSMO1 facilitates TAK1–TAB complex assembly to mediate activation of MAPK-p38 and NF-κB signaling

**Fig. 6:**
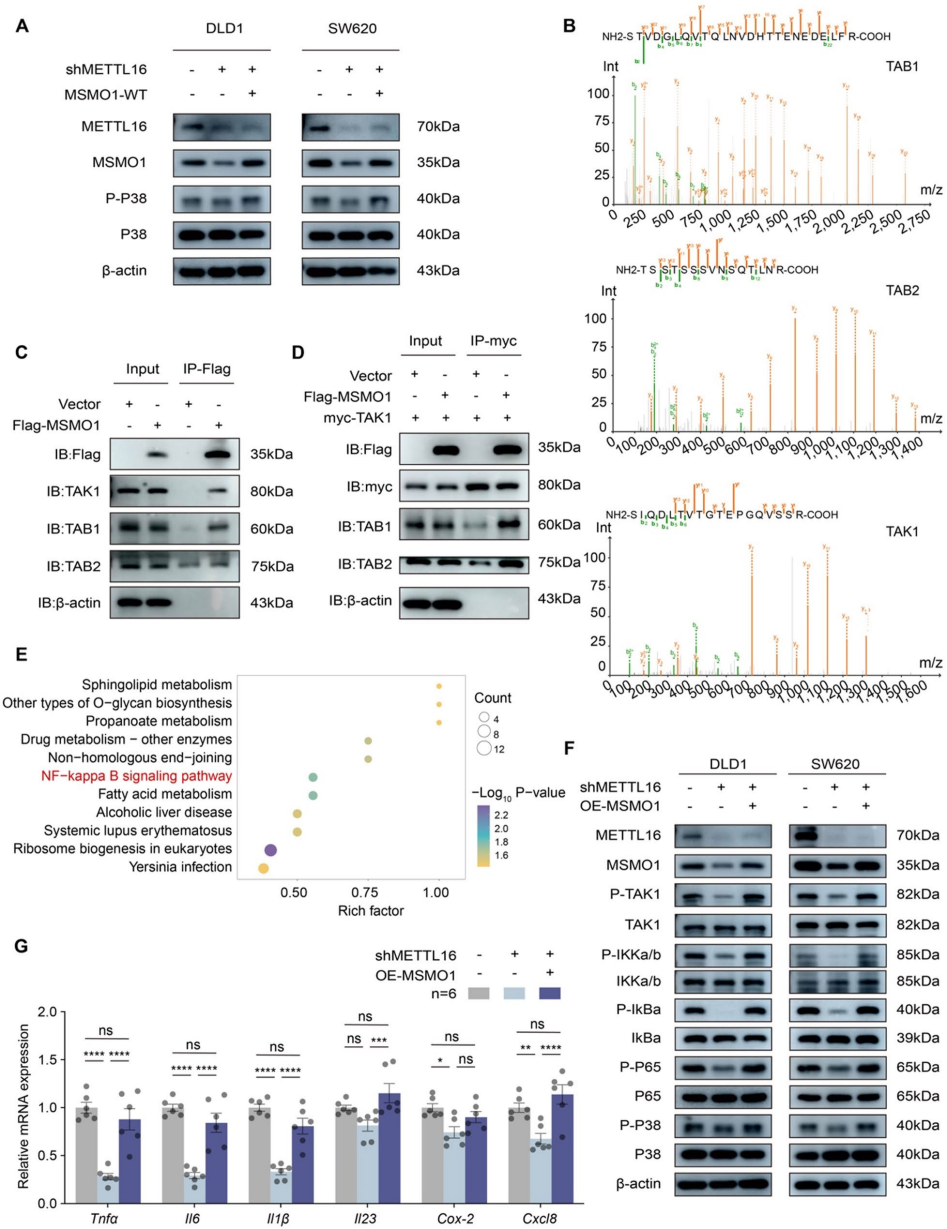
MSMO1 facilitates TAK1–TAB complex assembly to mediate activation of MAPK-P38 and NF-κB signaling. **A** Western blotting analysis showing MAPK-p38 pathway activity in DLD1 and SW620 cells following transfection with the indicated plasmids. **B** Representative mass spectrum of TAB1, TAB2, and TAK1. **C** 293T cells transfected with empty vector control or Flag-MSMO1 for 48 h were subjected to the co-IP assay. **D** 293T cells transfected with the vector control or Flag-MSMO1 and myc-TAK1 expression plasmids for 48 h were subjected to co-IP assay. **E** KEGG pathway enrichment of MSMO1-interacting proteins identified by immunoprecipitation, shown as a bubble plot of the top enriched pathways. **F** Analysis of the TAK1-MAPK/NFκB signaling pathway in the indicated stable cell lines by immunoblotting. **G** Relative mRNA expression levels of Tnfa, Il-6, Il-1β, Il-23, Cox-2 and Cxcl8 in DLD1 cells following transfection with the indicated plasmids. Results shown are the mean ± SEM (ns, non-significant; *p < 0.05, **p < 0.01, ***p < 0.001, ****p < 0.0001) of triplicate determination from three independent experiments

To elucidate whether METTL16 modulates the MAPK signaling cascade through MSMO1, we reintroduced MSMO1 into METTL16-deficient CRC cells, which effectively reinstated the phosphorylation of p38 MAPK **(**Fig. 6A**)**. To further clarify the molecular mechanism underlying MSMO1-mediated regulation of p38 activation, we performed immunoprecipitation coupled with mass spectrometry (IP-MS) to comprehensively characterize the MSMO1 interactome. This analysis identified 145 MSMO1-interacting proteins, among which several components of the TAK1–TAB signaling complex—specifically Tables 1 and 2, and TAK1—were recognized as potential interacting partners **(**Fig. 6B**)**. TAK1, a member of the MAP kinase kinase kinase (MAP3K) family, serves as a pivotal intracellular signaling node that orchestrates both the MAPK and NF-κB pathways, thereby regulating a broad spectrum of biological processes [34]. Co-immunoprecipitation (Co-IP) assays verified the physical interactions between MSMO1 and essential components of the TAK1–TAB complex, including TAK1, Table 1, and Table 2 **(**Fig. 6C**)**. The activation of TAK1 is known to rely on its association with TAB family adaptor proteins, wherein Table 1 predominantly binds to the N-terminal region of TAK1, while Tables 2 and 3 primarily associate with the C-terminal domain [35]. Notably, accumulating evidence indicates that Table 1 plays a central role in facilitating TAK1 autophosphorylation and achieving its full catalytic activation [36]. Flag-MSMO1 and Myc-TAK1 were co-transfected into 293T cells followed by Myc-IP. MSMO1 overexpression enhanced the interaction between TAK1 and Table 1/Table 2, suggesting a supportive role in complex formation (Fig. 6D). Consistently, KEGG pathway enrichment analysis of MSMO1-interacting proteins further highlighted the NF-κB signaling pathway (Fig. 6E). To evaluate the biological significance of the MSMO1–TAK1 interaction, TAK1 autophosphorylation and downstream signaling were assessed in shMETTL16 and shMETTL16 + MSMO1-OE CRC cells. METTL16 knockdown impaired TAK1 phosphorylation and reduced activation of MAPK-p38 and NF-κB pathways, as evidenced by decreased phosphorylation of p38, IKK, and IκBα, whereas MSMO1 overexpression restored these phosphorylation levels (Fig. 6F). In addition, NF-κB downstream pro-inflammatory mediators, including TNF-α, IL-6, IL-1β, IL-23, COX-2, and CXCL8, were downregulated upon METTL16 knockdown, but their expression was rescued by MSMO1 reintroduction (Fig. 6G). Taken together, these findings indicate that MSMO1 facilitates the assembly of the TAK1–Table 1/Table 2 complex, thereby promoting TAK1 autophosphorylation and activation, ultimately leading to enhanced MAPK-p38 and NF-κB signaling.

### METTL16/MSMO1 axis links cholesterol metabolism to ER stress and m^6^A regulation

**Fig. 7:**
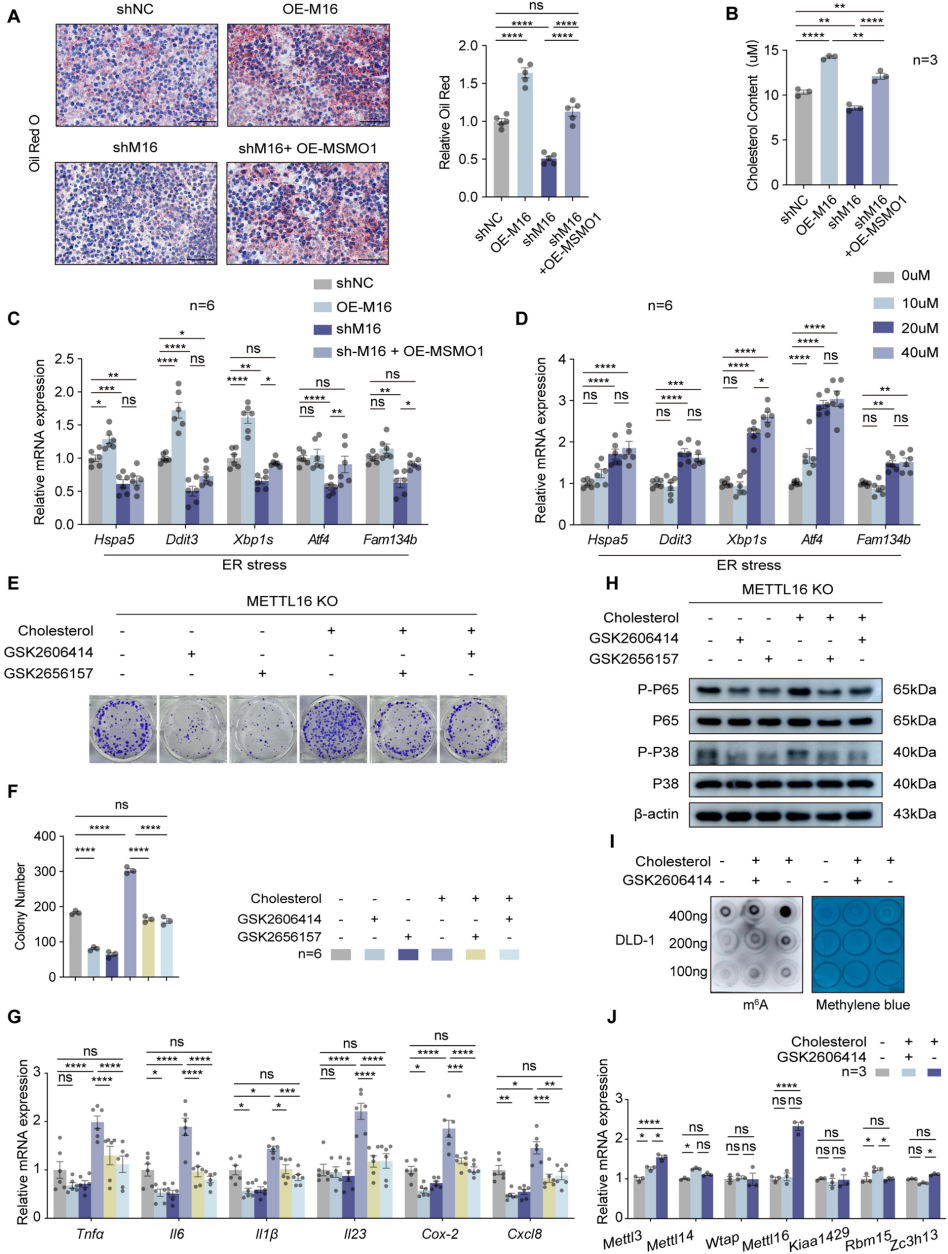
METTL16/MSMO1 axis links cholesterol metabolism to ER stress and m⁶A regulation. **A** Representative image of Oil Red O staining to examine lipid droplet distribution in Xenografts tumors derived from DLD1‐shNC, DLD1-OE-M16, DLD-shM16 and DLD1-shM16+OE-MSMO1. Scale bars=50 μm. Quantification of positive cells from Oil red O staining in xenografts tissues. **B** Cholesterol content was measured in DLD1 cells from shNC, OE-METTL16, shMETTL16, and shMETTL16+OE-MSMO1 groups. **C** Relative mRNA levels of Hspa5, Ddit3, Xbp1s, Atf4, Fam134b in DLD1 from shNC, OE-METTL16, shMETTL16, and shMETTL16+OE-MSMO1 groups. **D** Relative mRNA expression levels of Hspa5, Ddit3, Xbp1s, Atf4, Fam134b in DLD1 cells cultured under the indicated cholesterol concentrations. **E** and **F** Colony formation assays performed in DLD1 cells treated with cholesterol, GSK2606414, or GSK2656157 as indicated. Representative images (**E**) and quantification (**F**) of results were shown. **G** Relative mRNA expression levels of Tnfa, Il-6, Il-1β, Il-23, Cox-2 and Cxcl8 in DLD1 cells treated with cholesterol, GSK2606414, or GSK2656157 as indicated. **H** Western blotting analysis showing the phosphorylation levels of P65 and P38 in DLD1 cells treated with cholesterol, GSK2606414, or GSK2656157 as indicated. **I** m6A dot blot analysis showing total RNA m6A levels in DLD1 cells treated with cholesterol with or without GSK2606414. **J** Relative mRNA expression levels of m6A writers in DLD1 cells treated with cholesterol with or without GSK2606414. Results shown are the mean ± SEM (ns, non-significant; *p < 0.05, **p < 0.01, ***p < 0.001, ****p < 0.0001) of triplicate determination from three independent experiments

MSMO1, a key enzyme in cholesterol biosynthesis, catalyzes the first demethylation step of C4-methylsterols during the conversion of lanosterol to cholesterol [37]. Given its established metabolic function, alterations in MSMO1 expression are expected to directly influence intracellular cholesterol levels and sterol homeostasis. Accumulating evidence [18–20] indicates that dysregulated cholesterol metabolism contributes to CRC progression by promoting membrane synthesis, lipid raft–mediated signaling activation, and oncogenic pathway amplification. To assess the impact of the METTL16/MSMO1 axis on cellular lipid metabolism, we quantified lipid accumulation using Oil Red O staining. Knockdown of METTL16 significantly reduced cellular lipid content, while its overexpression led to increased lipid accumulation. Likewise, reintroduction of MSMO1 into METTL16-deficient cells resulted in elevated lipid levels **(**Fig. 7A**)**. These findings were further corroborated by measurements of cellular cholesterol levels, which exhibited a parallel trend **(**Fig. 7B**)**. Importantly, inhibition of p38 signaling with SB203580 did not affect intracellular cholesterol levels nor alter MSMO1 expression (Supplementary Fig. 6I and J), indicating that p38 activation does not regulate cholesterol biosynthesis.

Cholesterol is indispensable for maintaining membrane structural integrity, mediating intracellular signaling cascades, and sustaining essential cellular functions [38]. Aberrant lipid metabolism is tightly linked to endoplasmic reticulum (ER) stress, since excessive or unbalanced lipid—particularly cholesterol—accumulation perturbs ER membrane homeostasis and compromises its protein-folding capacity [39]. In malignant cells, elevated cholesterol biosynthesis not only fulfills the metabolic demands required for rapid proliferation but also provokes ER stress and activates adaptive signaling cascades, such as the unfolded protein response (UPR) [40, 41]. Based on these insights, we next explored whether the METTL16/MSMO1 regulatory axis modulates ER stress in CRC cells. The expression of key ER stress–related genes, including *Hspa5 Ddit3*, *Xbp1s*, *Atf4*, and *Fam134b*, was quantified using qRT-PCR. Silencing of METTL16 markedly attenuated ER stress–associated signaling, whereas re-expression of MSMO1 in METTL16-deficient cells restored and enhanced the expression of these ER stress markers **(**Fig. 7C**)**. Furthermore, treatment of CRC cells with escalating concentrations of exogenous cholesterol induced a dose-dependent increase in ER stress marker expression **(**Fig. 7D**)**. To elucidate the biological outcomes of cholesterol-induced ER stress, CRC cells were treated with cholesterol either alone or in combination with the ER stress inhibitors GSK2606414 and GSK2656157. Cholesterol supplementation markedly promoted cellular proliferation, whereas pharmacological inhibition of ER stress significantly attenuated this proliferative effect (Fig. 7E and F). Consistently, qRT-PCR analysis revealed that cholesterol exposure upregulated the mRNA expression of pro-inflammatory mediators, whereas blockade of ER stress signaling suppressed their transcriptional activation (Fig. 7G). Furthermore, Western blot analysis demonstrated that cholesterol treatment enhanced the phosphorylation of p38 and p65, while inhibition of ER stress elicited the opposite effect **(**Fig. 7H**)**.

Given that cholesterol metabolic reprogramming can trigger ER stress and inflammatory signaling cascades, we next investigated whether this metabolic perturbation also impacts epi-transcriptomic regulation. Notably, dot-blot assays demonstrated that cholesterol exposure led to an overall increase in global m^6^A methylation levels, whereas pharmacological inhibition of ER stress markedly diminished m^6^A modification **(**Fig. 7I**)**. Among m^6^A methyltransferases (“writers”), METTL3 and METTL16 expression was notably upregulated upon cholesterol stimulation; however, this induction was abolished when ER stress was pharmacologically inhibited **(**Fig. 7J**)**. A comparable expression pattern was also observed for the m^6^A readers (Supplementary Fig. 6K). Collectively, these findings highlight a reciprocal regulatory interplay between cholesterol metabolism and m^6^A modification, forming a metabolic–epi-transcriptomic feedback circuit that amplifies ER stress–mediated signaling in CRC cells.

## Discussion

N^6^-methyladenosine (m^6^A), the most prevalent internal modification of eukaryotic mRNA, exerts a critical regulatory influence on RNA metabolism by modulating key post-transcriptional processes, including pre-mRNA splicing, transcript stability, translational efficiency, and RNA decay [42, 43]. Among these, METTL16 functions as a multifunctional methyltransferase that modulates RNA stability, translation, and splice-site selection through its ability to bind and methylate specific RNA targets [27, 44–48]. The functional repertoire of METTL16 extends to the regulation of diverse cellular processes, including senescence, tumorigenesis, erythropoiesis, proliferation, and genome stability, thereby highlighting its pivotal role in preserving cellular homeostasis [10, 49–51]. Accumulating evidence suggests that METTL16 exhibits context-dependent dual functionality in tumorigenesis, acting as either a tumor promoter [12, 13, 52–54] or a suppressor [55–57], contingent upon the prevailing cellular and molecular milieu. These opposing effects are largely attributed to its multifunctionality and the intricate molecular regulatory mechanisms it engages. In this study, we demonstrate that METTL16 plays a critical oncogenic role in driving tumor progression in CRC. Although the regulatory functions of METTL3 and METTL14 in CRC pathogenesis are well established, comprehensive bioinformatic analyses reveal that METTL16 exhibits the strongest association with CRC progression among the METTL family of methyltransferases. METTL16 expression is markedly upregulated in CRC tissues and positively correlates with poor clinical prognosis, highlighting its potential utility as a diagnostic and prognostic biomarker for CRC. Furthermore, in vitro and in vivo functional assays demonstrate that METTL16 promotes CRC tumorigenesis through its m^6^A methyltransferase activity, thereby reinforcing its pivotal role in CRC progression.

Integrative analysis of RNA sequencing and MeRIP-seq data identified MSMO1 as a key downstream target of METTL16 in CRC. Validation in CRC cell lines further confirmed MSMO1 as a direct downstream effector of METTL16-mediated m^6^A modification. Mechanistically, METTL16 directly binds to *Msmo1* mRNA, catalyzes its m^6^A methylation, and stabilizes the transcript, thereby increasing MSMO1 protein abundance. MSMO1 is a key enzyme in the cholesterol biosynthetic pathway, functioning at the C4-demethylation step, where it catalyzes a three-step oxidative conversion of 4,4-dimethylsterols to their demethylated products, with T-MAS and dihydro-T-MAS serving as characteristic intermediates [37]. MSMO1 expression is finely regulated by intracellular sterol availability, and disruption of its enzymatic activity destabilizes cholesterol equilibrium, thereby altering membrane composition and provoking ER stress responses [37, 58]. Tumor cells commonly exhibit cholesterol metabolic reprogramming, typified by increased synthesis and uptake alongside reduced efflux, a metabolic adaptation closely linked to enhanced proliferation, migration, and therapeutic resistance [59, 60]. A study published in 2023 by Wei et al. [13] identified METTL16 as a pro-oncogenic factor in CRC, delineating a mechanistic cascade in which METTL16 promotes glycolytic reprogramming via m^6^A-dependent targeting of SOGA1, leading to AMPK complex ubiquitination and suppression, PDK4 upregulation, and enhanced glycolytic flux. Subsequently, a 2025 investigation further demonstrated that METTL16 drives lipid metabolic remodeling by stabilizing *Tm7sf2* mRNA through m^6^A modification in an IGF2BP1/2-dependent manner, thereby promoting lipid droplet accumulation and tumor progression [14]. Building upon this growing body of evidence, our study provides additional mechanistic insight into the relationship between METTL16 and cholesterol-associated metabolic control. Specifically, we observed that METTL16 regulates MSMO1 expression via m^6^A modification, which in turn influences intracellular cholesterol levels and ER stress pathway activity. METTL16 knockdown led to reduced MSMO1 abundance, accompanied by alterations in cholesterol metabolism and modulation of ER stress signaling. Importantly, restoration of MSMO1 expression effectively rescued these molecular and phenotypic changes, supporting the notion that MSMO1 represents a key downstream effector in this regulatory axis. These observations suggest that dysregulated cholesterol metabolism and ER stress responses may serve as important intermediates linking METTL16 activity to downstream oncogenic signaling events.

Moreover, integrated pathway enrichment analyses of RNA-seq datasets revealed significant changes in MAPK-related signaling, with p38 MAPK functioning as a central stress-responsive kinase involved in inflammatory transcriptional programs, apoptosis, autophagy, metabolic adaptation, and stemness-associated processes [61]. To further explore potential signaling connections downstream of MSMO1, mass spectrometry–based proteomic analyses indicated that MSMO1 may interact with the TAK1–Table 1/2 complex, thereby influencing activation of both MAPK/p38 and NF-κB/p65 pathways. In line with previous observations that cholesterol accumulation and ER stress can act as canonical stimuli for p38 activation [30, 62, 63], we propose that excessive sterol intermediates may increase ER membrane tension, oxidative stress, and mitochondrial metabolic burden, ultimately contributing to stress–inflammatory signaling activation. Supporting this model, supplementation of METTL16-deficient CRC cells with graded cholesterol concentrations, together with pharmacological modulation of ER stress, produced phenotypic and molecular alterations consistent with p38 and NF-κB pathway engagement. Taken together, our findings complement prior work on METTL16-mediated metabolic remodeling and further suggest a potential mechanistic link through which cholesterol homeostasis perturbation may be coupled to ER stress–associated inflammatory signaling during CRC progression.

Accumulating evidence [64] indicates that the tumor microenvironment (TME) is characterized by hypoxia, acidosis (lactate accumulation), nutrient deprivation, oxidative stress, and aberrant TCA cycle metabolites such as 2-HG, succinate, and fumarate. These metabolic perturbations influence RNA epi-transcriptomic regulation through two interconnected mechanisms: modulation of metabolite-derived cofactors required for methylation reactions and transcriptional reprogramming of RNA methylation machinery. On one hand, intracellular availability of SAM, the universal methyl donor for RNA methyltransferases, directly determines methylation capacity [45]. On the other hand, metabolic stress signaling pathways [65–68]—including HIF, mTOR, MYC, AMPK, and inflammatory cascades—reshape the “writer–eraser–reader” network, thereby dynamically remodeling RNA modification landscapes such as m^6^A, m^5^C, m^7^G, and ac^4^C.

Consistent with this metabolic–epi-transcriptomic crosstalk, recent studies have demonstrated that upstream transcription factors can regulate RNA methyltransferases. For example, Wei et al. (2023) [13] reported that YY1 suppresses METTL16 transcription by binding to its promoter, highlighting that METTL16 expression is subject to regulatory control rather than constitutive activity. In the present study, cholesterol loading significantly elevated global m^6^A levels in CRC cells, and this increase was partially reversed by inhibition of ER stress, suggesting that cholesterol metabolic perturbation functionally engages the m^6^A machinery. Several non–mutually exclusive mechanisms may underlie this phenomenon. First, cholesterol biosynthesis is closely linked to one-carbon metabolism and the methionine/ SAM cycle. As METTL16 functions as a SAM-responsive RNA methyltransferase involved in maintaining SAM homeostasis through regulation of MAT2A mRNA processing [45], cholesterol-driven metabolic changes may alter intracellular SAM availability, thereby modulating METTL16 activity and global m^6^A deposition. Second, cholesterol accumulation can activate sterol-responsive and inflammatory signaling pathways, including SCAP–SREBP2 and NF-κB [69], which may indirectly reshape transcriptional programs governing m^6^A-related enzymes. Third, excessive cholesterol induces ER stress and unfolded protein response signaling, processes that have been implicated in dynamic remodeling of the m^6^A landscape [70]. Although the precise upstream mechanism linking cholesterol metabolism to METTL16 upregulation remains to be defined, our findings might provide a new perspective on the role of cholesterol metabolism in tumorigenesis, particularly in the context of epigenetic regulation via RNA methylation.

**Fig. 8:**
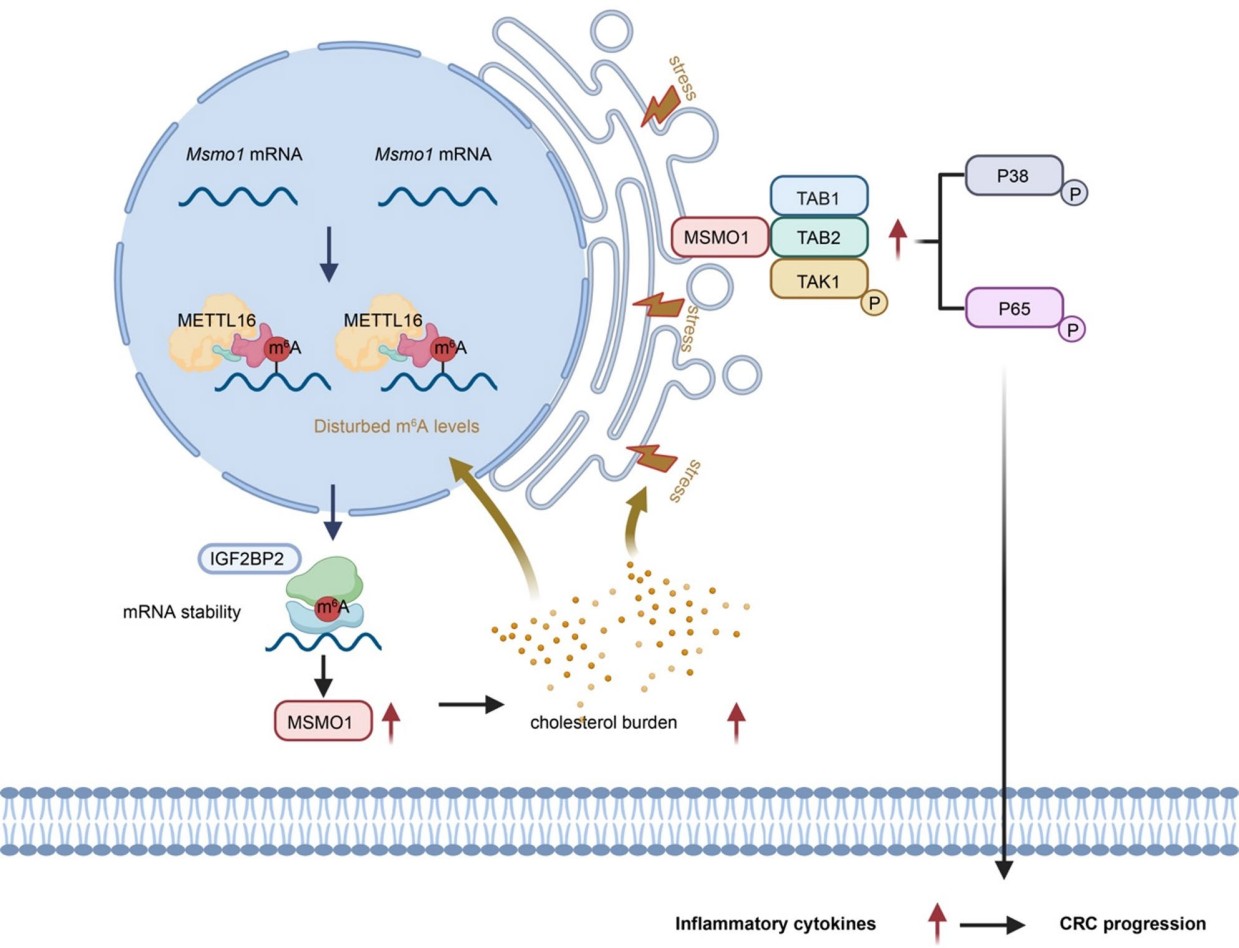
Schematic illustration depicting the proposed mechanism by which METTL16-mediated m^6^A modification enhances MSMO1 expression, leading to increased intracellular cholesterol levels, which induce ER stress and subsequently promote activation of the TAK1/TAB complex and downstream p38–p65 signaling

In summary, METTL16 expression is markedly elevated in CRC issues and correlates with an unfavorable clinical prognosis. Mechanistically, METTL16 facilitates m^6^A modification that enhances *Msmo1* transcript stability and expression through an IGF2BP2-dependent mechanism, which consequently alters intracellular cholesterol homeostasis, thereby inducing ER stress and activating the downstream MAPK-p38 and NF-κB signaling cascade, ultimately driving tumor progression. Moreover, elevated cholesterol levels were observed to influence both the global methylation landscape and the expression profiles of methyltransferases in CRC cells, suggesting the existence of a regulatory feedback loop between the cholesterol metabolic microenvironment and the epigenetic modification network of tumor cells **(**Fig. 8**)**. Collectively, these findings provide novel insights into potential diagnostic biomarkers and therapeutic targets for CRC.

## Supplementary Information

Supplementary Material 1.

Supplementary Material 2.

Supplementary Material 3.

Supplementary Material 4.

Supplementary Material 5.

Supplementary Material 6.

Supplementary Material 7.

Supplementary Material 8.

## Data Availability

All data needed to evaluate the conclusions in the paper are present in the main text and/or the Supplementary information. Further data supporting the findings of this study is available upon reasonable request. Please contact the corresponding author.

## Abbreviation

ALKBH5: AlkB homolog 5, RNA demethylase
ATF4: Activating transcription factor 4
BCAT1: Branched-chain amino acid transaminase 1
BCAT2: Branched-chain amino acid transaminase 2
COX-2: Cyclooxygenase-2
CRC: Colorectal cancer
CXCL8: C-X-C motif chemokine ligand 8
DDIT3: DNA damage inducible transcript 3 (CHOP)
EGFR: Epidermal growth factor receptor
ER: Endoplasmic reticulum
FAM134B: Family with sequence similarity 134 member B
FTO: Fat mass and obesity-associated protein
HSPA5: Heat shock protein family A member 5 (GRP78/BiP)
IGF2BP: Insulin-like growth factor 2 mRNA-binding protein
IKK: IκB kinase
IL-1β: Interleukin-1 beta
IL-6: Interleukin-6
IL-23: Interleukin-23
KRAS: Kirsten rat sarcoma viral oncogene homolog
LXR: Liver X receptor
MAPK: Mitogen-activated protein kinase
m^6^A: N^6^-methyladenosine
METTL3: Methyltransferase-like protein 3
METTL14: Methyltransferase-like protein 14
METTL16: Methyltransferase-like protein 16
MSMO1: Methylsterol monooxygenase 1
NF-κB: Nuclear factor kappa-light-chain-enhancer of activated B cells
p38: p38 mitogen-activated protein kinase
p65: NF-κB p65 (RELA) subunit
PI3K: Phosphoinositide 3-kinase
SAM: S-adenosylmethionine
SC4MOL: Sterol-C4-methyl oxidase-like (alternative name for MSMO1)
SCD1: Stearoyl-CoA desaturase 1
TAB1/2: TAK1-binding protein 1/2
TAK1: Transforming growth factor β-activated kinase 1 (MAP3K7)
T-MAS: T-methylated sterol intermediate
TNF-α: Tumor necrosis factor alpha
UPR: Unfolded protein response
XBP1s: Spliced X-box binding protein 1
