# Supplementary figures and images for: METTL16-mediated m6A modification of MSMO1 modulates cholesterol metabolism and activates MAPK-p38/NF-κB signaling in colorectal cancer
Source: J Exp Clin Cancer Res. 2026 Mar 17;45:121. doi: 10.1186/s13046-026-03690-x (PMC13181995; doi:10.1186/s13046-026-03690-x)

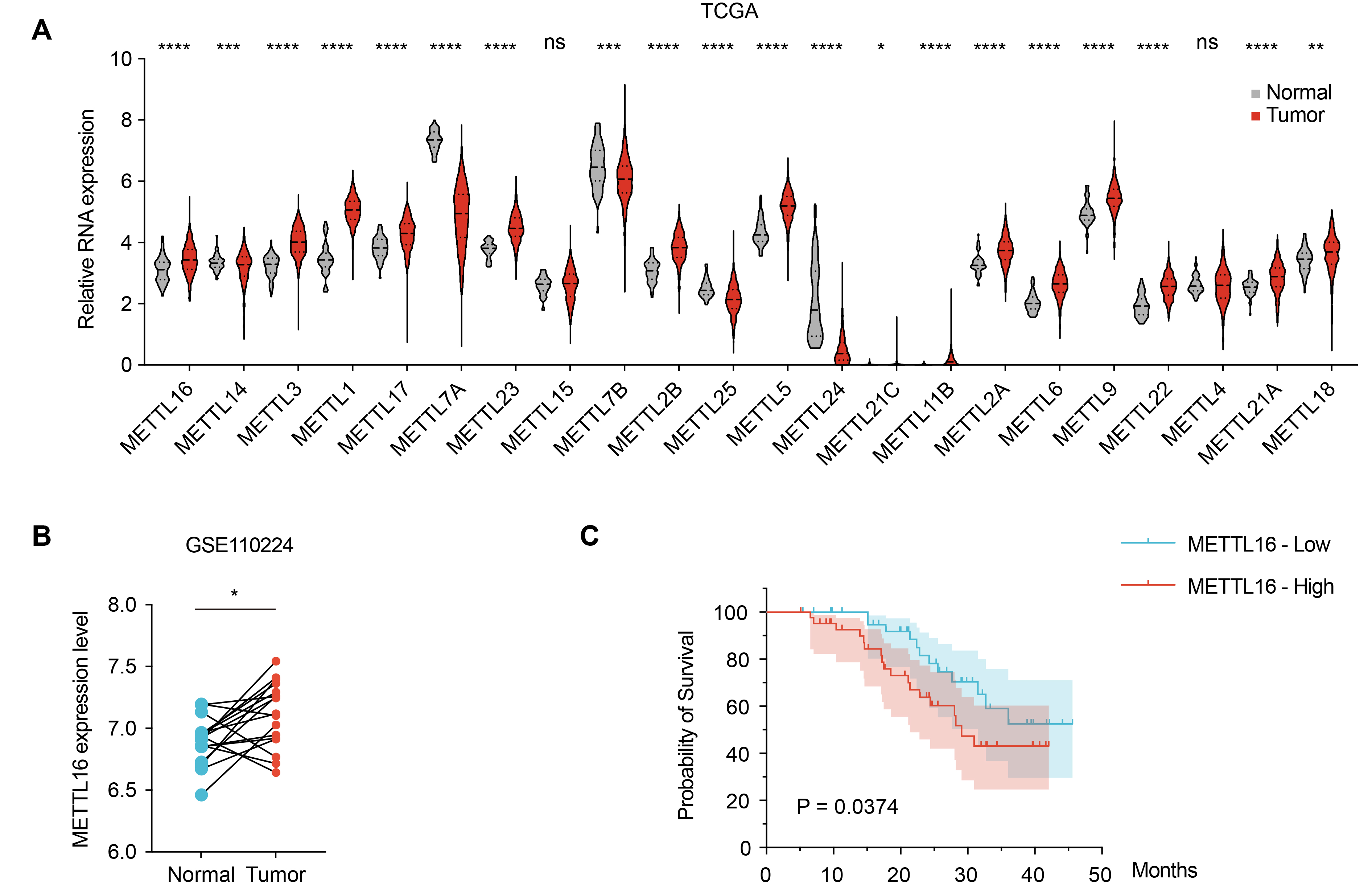

Supplement: Supplementary file 1 — Supplementary Material 1. [file 13046_2026_3690_MOESM1_ESM.jpg]

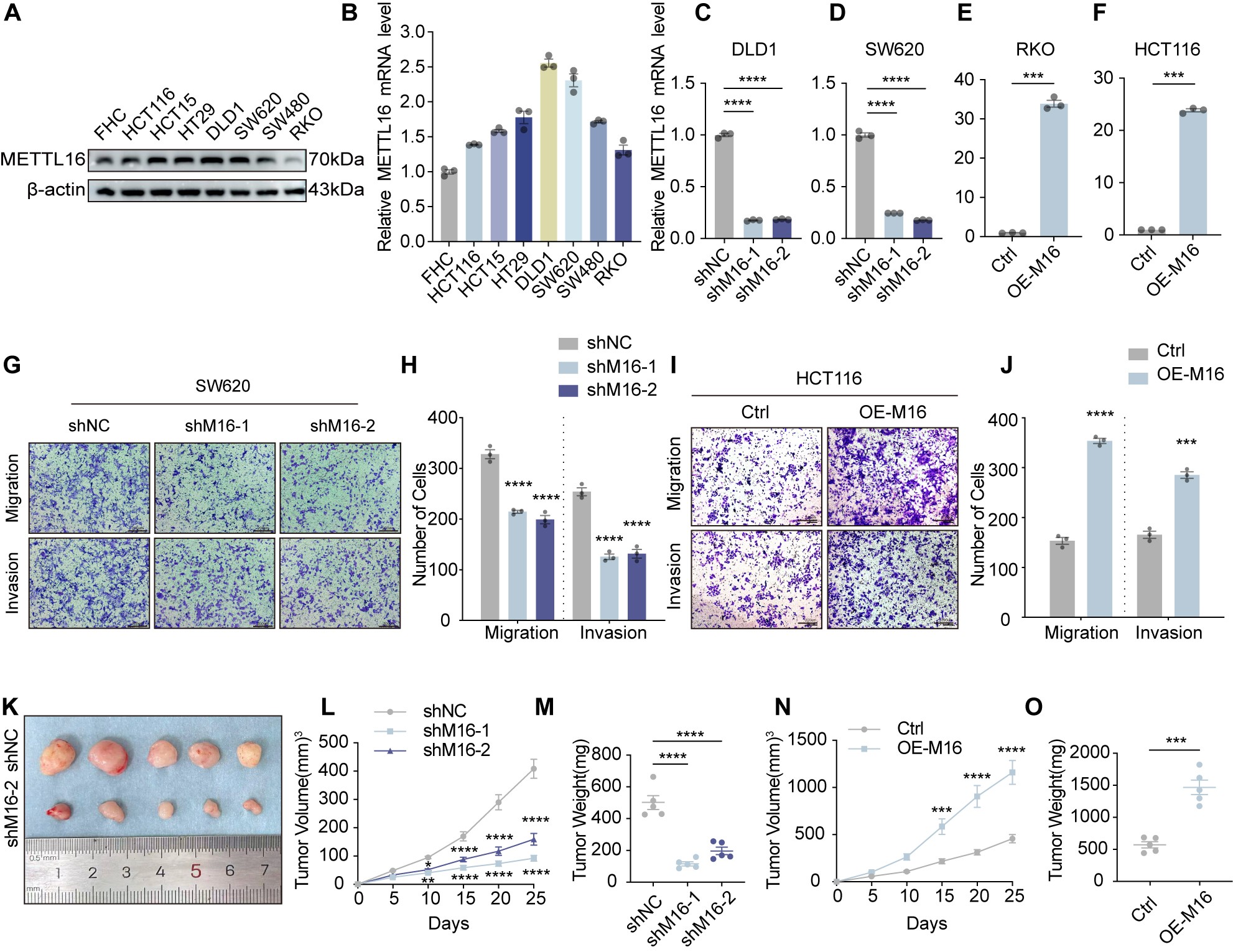

Supplement: Supplementary file 2 — Supplementary Material 2. [file 13046_2026_3690_MOESM2_ESM.jpg]

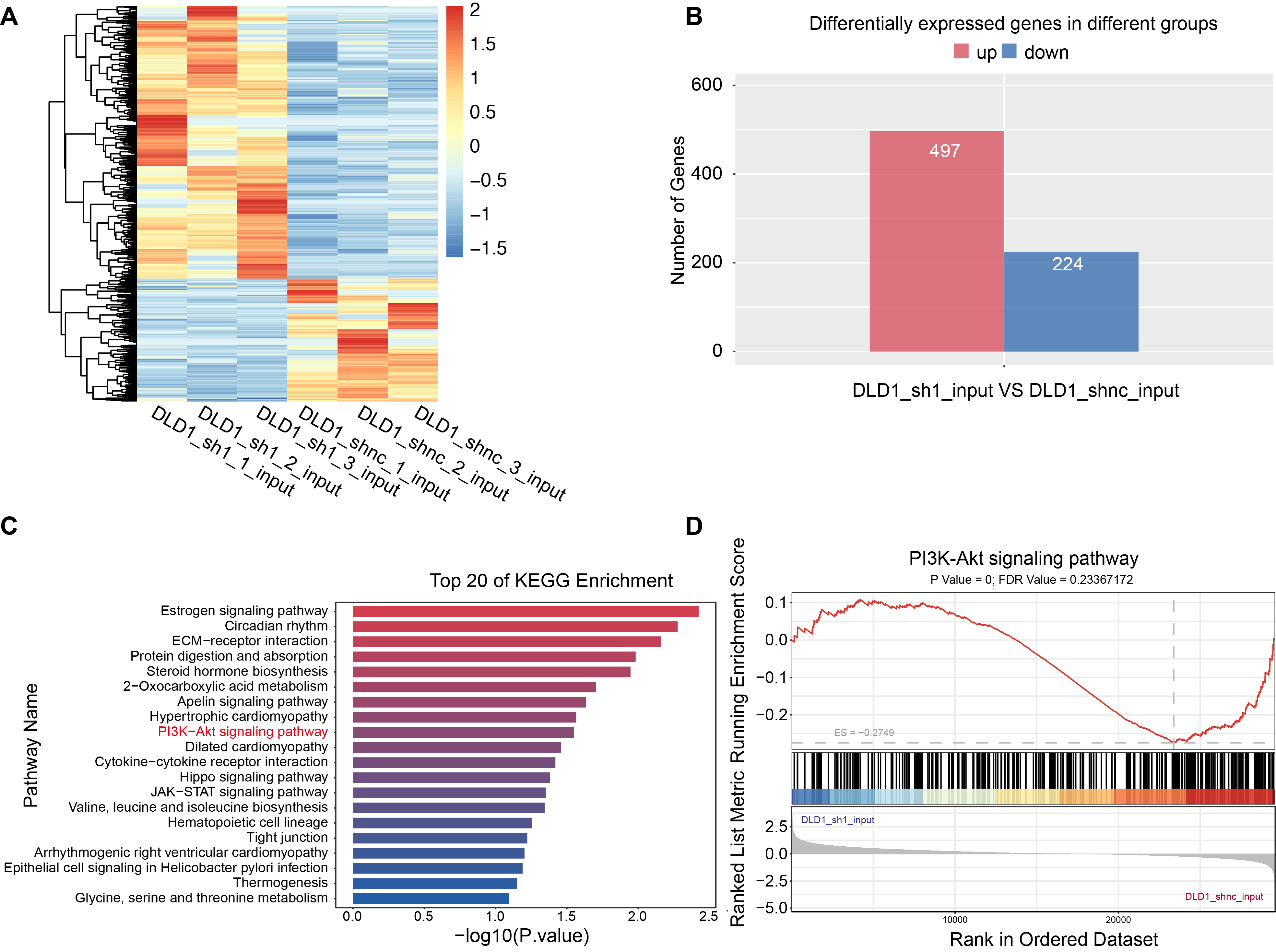

Supplement: Supplementary file 3 — Supplementary Material 3. [file 13046_2026_3690_MOESM3_ESM.jpg]

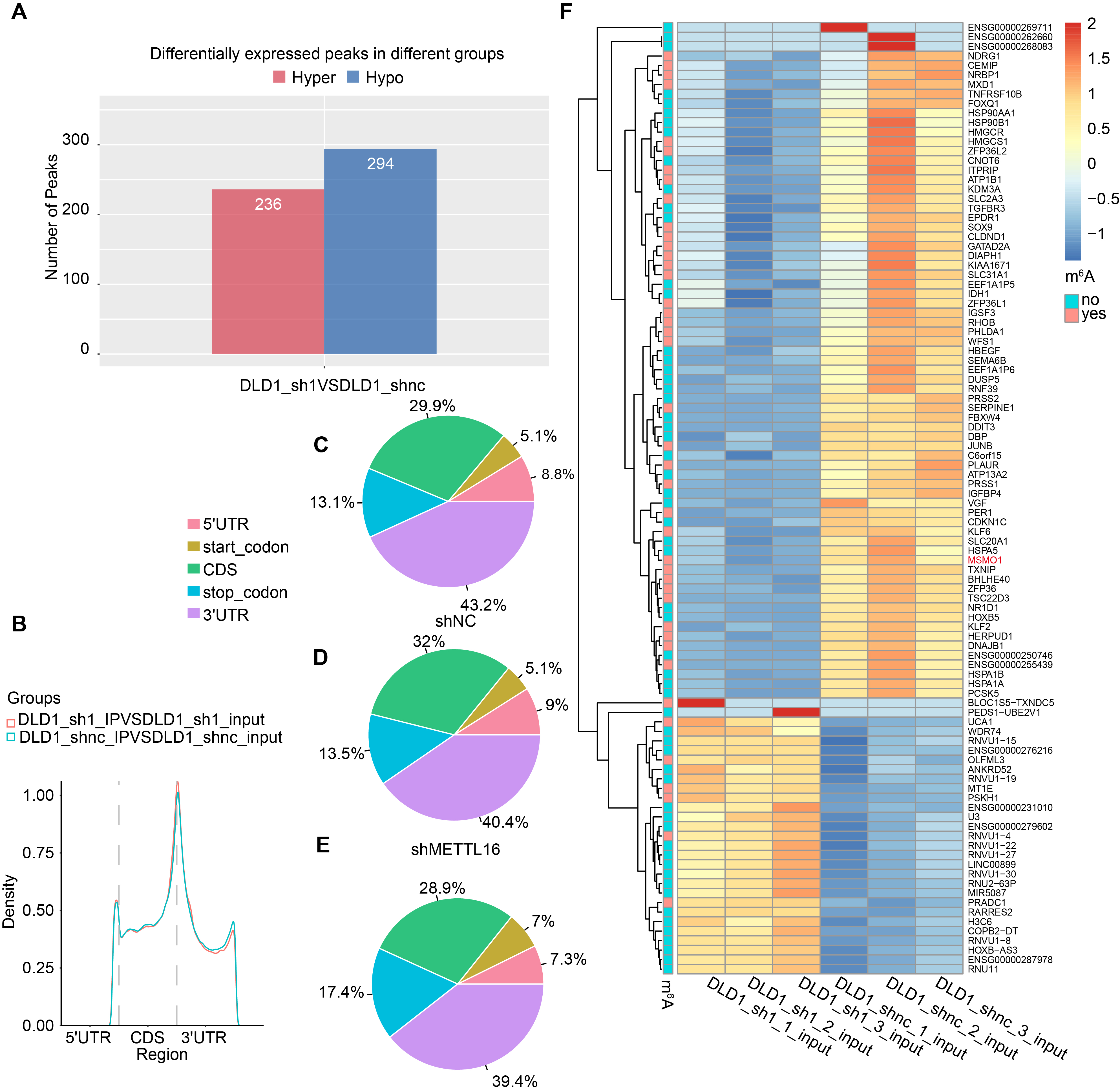

Supplement: Supplementary file 4 — Supplementary Material 4. [file 13046_2026_3690_MOESM4_ESM.jpg]

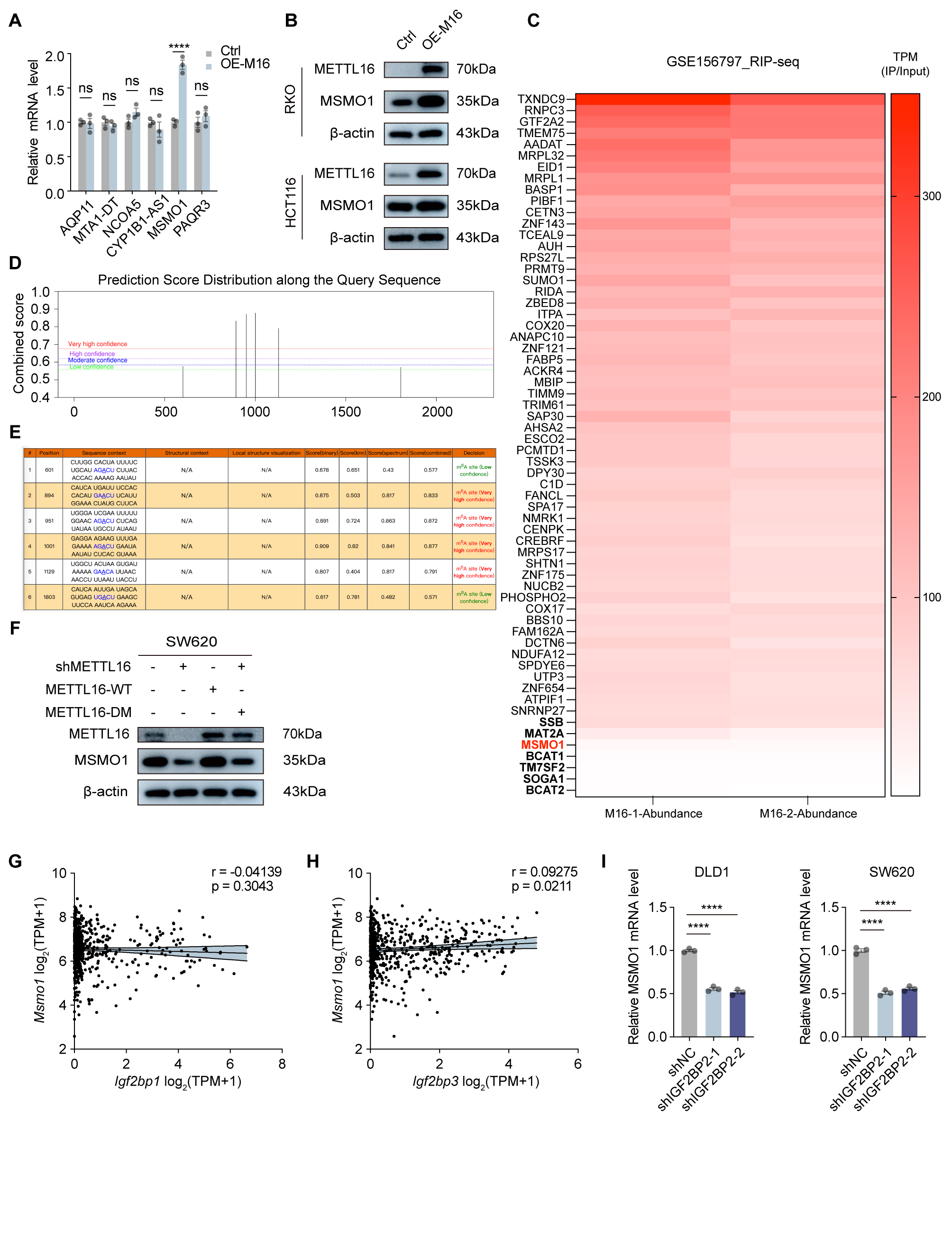

Supplement: Supplementary file 5 — Supplementary Material 5. [file 13046_2026_3690_MOESM5_ESM.jpg]

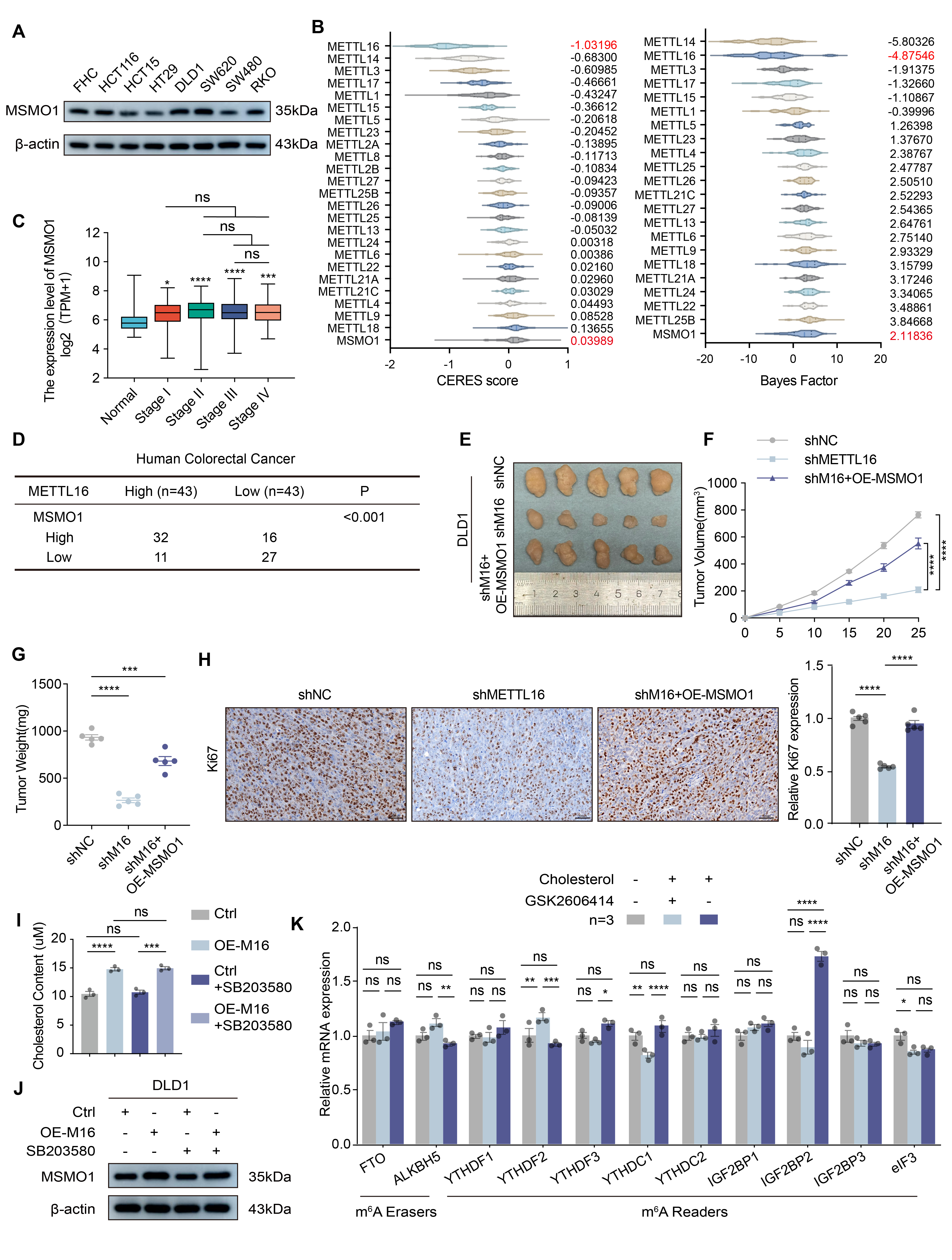

Supplement: Supplementary file 6 — Supplementary Material 6. [file 13046_2026_3690_MOESM6_ESM.jpg]
