# Supplementary material for: METTL16-mediated m6A modification of MSMO1 modulates cholesterol metabolism and activates MAPK-p38/NF-κB signaling in colorectal cancer
Source: J Exp Clin Cancer Res. 2026 Mar 17;45:121. doi: 10.1186/s13046-026-03690-x (PMC13181995; doi:10.1186/s13046-026-03690-x)
Supplement: Supplementary file 7 — Supplementary Material 7. [file 13046_2026_3690_MOESM7_ESM.docx]

**METTL16-Mediated m^6^A Modification of MSMO1 Modulates Cholesterol Metabolism and Activates MAPK-p38/NF-κB Signaling in Colorectal Cancer**

Yongheng Zhao^1†^, Tingyue Gong^1†^, Hao Li^1†^, Haiping Lin^3^, Minhao Yu^1^, Yang Luo^1^**^*^**, Ming Zhong^1,2^**^*^** Jun Qin^1^**^*^**

*^1^ Department of* *Gastrointestinal Surgery, Renji Hospital Affiliated to Shanghai Jiao Tong University School of Medicine, Shanghai, China*

*^2^* *Department of General Surgery, Shanghai Pudong New Aera Gongli Hospital, Shanghai, China*

*^3^Department of General Surgery, Jinhua Central Hospital, Teaching Hospital of Mathematical Medicine College, Zhejiang Normal University, Zhejiang, China*

†These authors contributed equally to this work.

*Corresponding authors:

Jun Qin, Department of Gastrointestinal Surgery, Renji Hospital, School of Medicine, Shanghai Jiao Tong University, Shanghai, China. E-mail: [qinjun@renji.com](mailto:qinjun@renji.com)

Ming Zhong, Department of General Surgery, Shanghai Pudong New Area Gongli Hospital, Shanghai, China. E-mail: [drzhongming1966@163.com](mailto:drzhongming1966@163.com)

Yang Luo, Department of Gastrointestinal Surgery, Renji Hospital, School of Medicine, Shanghai Jiao Tong University, Shanghai, China. E-mail: [lykshuiyang@163.com](mailto:lykshuiyang@163.com)
