# Supplementary material for: METTL16-mediated m6A modification of MSMO1 modulates cholesterol metabolism and activates MAPK-p38/NF-κB signaling in colorectal cancer
Source: J Exp Clin Cancer Res. 2026 Mar 17;45:121. doi: 10.1186/s13046-026-03690-x (PMC13181995; doi:10.1186/s13046-026-03690-x)
Supplement: Supplementary file 8 — Supplementary Material 8. [file 13046_2026_3690_MOESM8_ESM.docx]

**Table 1** Antibodies used in the experiment

| Antibody | Lot | Company |
| --- | --- | --- |
| m6A | ab284130 | Abcam |
| METTL16 | ab313743 | Abcam |
| MSMO1 | 84742-1-RR | Proteintech |
| MEK1/2 | T55168 | Abmart |
| p-MEK1/2 (Ser218/222/226) | TA8035 | Abmart |
| ERK1/2 | 4695 | CST |
| p-ERK (Thr202/Tyr204) | 4370 | CST |
| JNK | 9252 | CST |
| p-JNK (Thr183/Tyr185) | 4668 | CST |
| AKT1 | 2938 | CST |
| p-AKT1 (Thr184/187) | 9018 | CST |
| P38 | 8690 | CST |
| p-p38 (Thr180/182) | 4511 | CST |
| IKKa/b | T55660F | Abmart |
| p-IKKa/b (Ser176/177) | TP56290F | Abmart |
| IkBa | 4814 | CST |
| p-IkBa (Ser32) | 2859 | CST |
| NF-κB | 8242 | CST |
| Phospho-NF-κB (Ser536) | 3031 | CST |
| TAB1 | 27566-1-AP | Proteintech |
| TAB2 | 14410-1-AP | Proteintech |
| TAK1 | 5206 | CST |
| p-TAK1 (Thr184/187) | 4508 | CST |
| IGF2BP2 | 82757-2-RR | Proteintech |
| Beta-Actin | 66009-1-Ig | Proteintech |
| Flag | F1804 | Sigma |
| Myc | M20002 | Abmart |
| HRP-conjugated Goat anti-Rabbit IgG (H+L) | AS014 | Abclonal |
| HRP-conjugated Goat anti-Mouse IgG (H+L) | AS003 | Abclonal |
| Anti-Rabbit Mouse IgG-HRP | M21009 | Abmart |
| IgG | A7016 | Beyotime |

**Table 2** Primers, shRNA used in the experiment

| Gene | Forwards (5’-3’) | Reverse (5’-3’) |
| --- | --- | --- |
| METTL16  MSMO1  MSMO1(MeRIP)  PAQR3  AQP11  NCOA5  CYP1B1-AS1  MTA1-DT  IGF2BP2  β-actin  GAPDH  TNF-a  IL-6  IL-1b  IL-23  COX-2  CXCL8  HSPA5  DDIT3  XBP1s  ATF4  FAM134B  METTL3  METTL14  WTAP  KIAA1429  RBM15  ZC3H13  FTO  ALKBH5  YTHDF1  YTHDF2  YTHDF3  YTHDC1  YTHDC2  IGF2BP1  IGF2BP3  EIF3c | TGGAGCAACCTTGAATGGCTGG  GCTGCCTTTGATTTGTGGAACCT  TTCTATGCTGGTTCTCGGCA  CACTGGGTTTGGCTCAATGGAG  CACTTCCAGGAAGTCCGAACCA  AGATTCACCGCTCCTGCACAGT  GGAAACTGGGAGATCTGCTGTG  AGTCCAGTGAGTGACCCAGA  GTTGGTGCCATCATCGGAAAGG  CACCATTGGCAATGAGCGGTTC  GTCTCCTCTGACTTCAACAGCG  CTCTTCTGCCTGCTGCACTTTG  AGACAGCCACTCACCTCTTCAG  CCACAGACCTTCCAGGAGAATG  GAGCCTTCTCTGCTCCCTGATA  CGGTGAAACTCTGGCTAGACAG  GAGAGTGATTGAGAGTGGACCAC  CTGTCCAGGCTGGTGTGCTCT  GGTATGAGGACCTGCAAGAGGT  CTGCCAGAGATCGAAAGAAGGC  TTCTCCAGCGACAAGGCTAAGG  GTCTCAGAGGTATCCTGGACTG  CTATCTCCTGGCACTCGCAAGA  CTGAAAGTGCCGACAGCATTGG  GCAACAACAGCAGGAGTCTGCA  TGACCTTGCCTCACCAACTGCA  CTTCCCACCTTGTGAGTTCTCC  CGGACAGTGATGCCTACAACAG  CCAGAACCTGAGGAGAGAATGG  CCAGCTATGCTTCAGATCGCCT  CAAGCACACAACCTCCATCTTCG  TAGCCAGCTACAAGCACACCAC  GCTACTTTCAAGCATACCACCTC  TCAGGAGTTCGCCGAGATGTGT  GGTTCTACTGGCAAGTCAGCCA  CTTTGTAGGGCGTCTCATTGGC  TCGTGACCAGACACCTGATGAG  GGAAGTGCCTGGACTGCATCAA | CCATCAGGAGTGTCTTCTGTGG  CTGCACAACCAAAGCATCTTGCC  TTCTGCTCTCCAGAAGCAATGT  AGTACCGCTCTGGGACTTTGGA  GTAGCGAAAGTGCCAAAGCTGG  CTGTCTGGCAATCTCCTCACGT  GGTTCTGTTCCAACCCTACCTC  CTGTGTGCTGGATTCCCCAT  TGGATGGTGACAGGCTTCTCTG  AGGTCTTTGCGGATGTCCACGT  ACCACCCTGTTGCTGTAGCCAA  ATGGGCTACAGGCTTGTCACTC  TTCTGCCAGTGCCTCTTTGCTG  GTGCAGTTCAGTGATCGTACAGG  GACTGAGGCTTGGAATCTGCTG  GCAAACCGTAGATGCTCAGGGA  CACAACCCTCTGCACCCAGTTT  CTTGGTAGGCACCACTGTGTTC  CTTGTGACCTCTGCTGGTTCTG  CTCCTGGTTCTCAACTACAAGGC  CTCCAACATCCAATCTGTCCCG  TTCCTCACTGGGTCGGTCAAGA  GCTTGAACCGTGCAACCACATC  CTCTCCTTCATCCAGATACTTACG  CTGCTGGACTTGCTTGAGGTAC  AGCAACCTGGTGGTTTGGCTAG  CTTCTTGTTCTCATACCTAACTCC  TCTGTGAGGTGCGAGGGACTAA  CGATGTCTGTGAGGTCAAACGG  GGTTCTCTTCCTTGTCCATCTCC  GTAAGAAACTGGTTCGCCCTCAT  CAACCGTTGCTGCAGTCTGTGT  ACAGGACATCTTCATACGGTTATTG  AGGATGGTGTGGAGGTTGTTCC  GGTTCTACTGGCAAGTCAGCCA  CCTTCACAGTGATGGTCCTCTC  GGTGCTGCTTTACCTGAGTCAG  CCATTCGTTCCACCAGAGTTAGG |

| Oligonucleotides | Sequences |  |
| --- | --- | --- |
| shMETTL16-1 | CCCTTGAGACTCAACTATATT |  |
| shMETTL16-2 | ATGGCTGGTATTTCCTCGCAA |  |
| shMSMO1-1 | TGATTTGTGGAACCTATTATT |  |
| shMSMO1-2 | GCATAGACTCTTACACCACAA |  |
| shIGF2BP2-1 | CAUGCCGCAUGAUUCUUGATT |  |
| shIGF2BP2-2 | GAACGAACUGCAGAACUUATT |  |

**Table 3** Correlation between METTL16 expression and different clinical characteristics

| Characteristics | n = 86 | High (%)  (n = 43） | Low (%)  (n = 43) | χ^2^ | P |
| --- | --- | --- | --- | --- | --- |
| Age  <60  ≥60  Gender  Male  Female  T stage  T1-T2  T3-T4  N stage  N0  N1, N2 | 29 (33.72%)  57 (66.28%)  48 (55.81%)  38 (44.19%)  22 (25.58%)  64 (74.42%)  52 (60.47%)  34 (39.53%) | 14(32.56%)  29(67.44%)  25(58.14%)  18(41.86%)  8(18.60%)  35(81.40%)  23(53.49%)  20(46.51%) | 15(34.88%)  28(65.12%)  23(53.49%)  20(46.51%)  14(32.56%)  29(67.44%)  29(67.44%)  14(32.56%) | 0.052  0.189  3.087  1.751 | 0.820  0.664  0.079  0.186 |
| M stage  M0  M1 | 73 (84.88%)  13 (15.12%) | 34(79.07%)  9(20.93%) | 39(90.70%)  4(9.30%) | 2.266 | 0.132 |
| TNM stage |  |  |  | 3.903 | 0.048 |
| I-II | 51 (59.30%) | 21(48.84%) | 30(69.77%) |  |  |
| III-IV | 35 (40.70%) | 22(51.16%) | 13(30.23%) |  |  |
